# Obesity Subphenotypes in Atrial Fibrillation: A Linked Electronic Health Record Study in the United Kingdom

**DOI:** 10.1016/j.jacadv.2026.103103

**Published:** 2026-08-05

**Authors:** Hiroyuki Yoshimura, Chang Xu, Gregory Y.H. Lip, Floriaan Schmidt, Rui Providencia

**Affiliations:** a Institute of Health Informatics Research, University College London, London, United Kingdom; b Liverpool Centre for Cardiovascular Science at University of Liverpool, Liverpool John Moores University and Liverpool Heart & Chest Hospital, Liverpool, United Kingdom; c Department of Clinical Medicine, Aalborg University, Aalborg, Denmark; d Department of Cardiology, Lipidology and Internal Medicine with Intensive Coronary Care Unit, Medical University of Bialystok, Bialystok, Poland; e Institute of Cardiovascular Science, Faculty of Population Health, University College London, London, United Kingdom; f UCL British Heart Foundation Center of Excellence, London, United Kingdom; g Department of Cardiology, Amsterdam Cardiovascular Sciences, Amsterdam University Medical Centre, University of Amsterdam, Amsterdam UMC, Amsterdam Zuidoost, the Netherlands; h Barts Heart Centre, Barts Health NHS Trust, London, United Kingdom

**Keywords:** all-cause mortality, atrial fibrillation, BMI, body mass index, obesity paradox, heart failure

## Abstract

**Background:**

Atrial fibrillation (AF) and obesity are increasingly prevalent and interrelated, but how body mass index (BMI) influences cardiovascular outcomes and mortality in AF patients at a population level remains uncertain.

**Objectives:**

This study aimed to assess cardiovascular hospitalizations and mortality among AF patients by BMI.

**Methods:**

Using linked Clinical Practice Research Datalink, Hospital Episodes Statistic, and Office for National Statistics data, we identified 71,519 adults with newly diagnosed AF (1998-2016) and a BMI measure within 12 months of diagnosis. Patients were grouped by World Health Organization BMI categories: underweight, normal weight, overweight, and obese. Primary outcome was all-cause mortality; secondary outcomes were hospitalizations for heart failure (HF), myocardial infarction, ischemic stroke, and bleeding.

**Results:**

The mean age was 74.9 ± 11.3 years; 44.9% were females. Over the median follow-up 5.72 years, 32,198 patients (45.0%) died. After multivariable adjustment, underweight was associated with higher all-cause mortality (HR: 1.87; 95% CI: 1.77-1.96), whereas overweight (HR: 0.72; 95% CI: 0.70-0.74) and obese (HR: 0.69; 95% CI: 0.67-0.71) categories had lower mortality vs normal weight. Obesity was independently associated with increased HF hospitalization risk, whereas overweight was modestly protective. Obesity was associated with lower adjusted myocardial infarction and bleeding hospitalization and showed no significant adjusted association with ischemic stroke. Cause-specific mortality analysis showed higher circulatory and neoplasm deaths among obese patients and higher respiratory deaths in underweight patients.

**Conclusions:**

In this large, nationwide AF cohort, being underweight at the time of AF diagnosis was associated with higher mortality, whereas being overweight and obese were paradoxically associated with lower all-cause mortality despite increased HF hospitalizations in obese patients.

Atrial fibrillation (AF) is the most common sustained arrhythmia, affecting over 50 million people worldwide.^1^^,^^2^ AF is associated with markedly increased risks of stroke, heart failure (HF), myocardial infarction, and all-cause mortality.^2^^,^^3^ An aging population has driven a parallel rise in AF and its complications, raising public health concerns and increasing health care costs in Western countries.^4^

Concurrently, global obesity rates have risen over recent decades.^5^^,^^6^ Overweight and obesity substantially increase the risk of cardiovascular disease, including AF.^7^ Higher adiposity frequently coexists with hypertension, dyslipidemia, and diabetes—conditions that can independently and synergistically elevate cardiovascular risk.^8^ The relationship between obesity and cardiovascular disease is therefore multifactorial and influenced by interactions with other established risk factors.^9^ For patients with AF receiving oral anticoagulation (OAC), bleeding remains one of the major complications. Previous studies have reported that both low and high body mass index (BMI) are associated with an increased risk of bleeding among AF patients treated with OAC.^10^

AF develops from a complex interplay of risk factors and comorbidities, many of which are worsened by obesity (eg, hypertension and obstructive sleep apnea).^11^ BMI is the most commonly used measure of adiposity.^12^ The World Health Organization classifies BMI as underweight (<18.5 kg/m^2^), normal weight (18.5-24.9 kg/m^2^), overweight (25-29.9 kg/m^2^), and obese (≥30 kg/m^2^).^13^

Nevertheless, knowledge is limited on the distinct health trajectories of AF patients across BMI categories. A better understanding of how varying degrees of adiposity affect AF progression and survival could improve risk stratification and inform the allocation of health care resources and prevention efforts. We aimed to evaluate cardiovascular hospitalizations and mortality among AF patients in the United Kingdom, stratified by the four World Health Organization BMI categories.

## Methods

We investigated the cardiovascular hospitalization events and death in patients with AF, stratified by BMI categories, using nationwide UK electronic health record data set. Individuals with newly diagnosed AF between January 1, 1998, and May 31, 2016, were identified, provided BMI data were available at the time of diagnosis. In addition, we examined the distribution of causes for death, classified according to International Classification of Diseases tenth revision chapters, across BMI categories.

### Data sources

The study data were linked from primary care (Clinical Practice Research Datalink [CPRD]), secondary care (Hospital Episodes Statistic), and national mortality records (Office for National Statistics), comprising a total of 6,529,382 participants.^2^ This data set includes data from approximately 10% of UK general practices and covers England, Scotland, Wales, and Northern Ireland. This data set is broadly representative of the UK population with respect to age, sex, socioeconomic diversity, geographic location, and prevalence and management of major health conditions. Previous studies have demonstrated its accuracy, reliability and completeness.^14^, ^15^, ^16^

### Study design

The study period extended from January 1, 1998, to May 31, 2016. The index date (study entry) was defined as the date of first recorded AF diagnosis. Follow-up continued until the earliest occurrence of death, transfer out of the general practice, or the end of the study period (May 31, 2016).

The primary outcome was all-cause mortality during the follow-up. Secondary outcomes included cardiovascular hospitalizations for myocardial infarction, HF, ischemic stroke, and bleeding.

Demographic variables included age at AF diagnosis (in years), sex, and ethnicity (categorized as White or non-White). Lifestyle factors comprised smoking status (never smoker vs current or former smoker), and alcohol-related disorders. Socioeconomic status was assessed using the Index of Multiple Deprivation, with the most deprived quintile compared against all others). Relevant clinical comorbidities present at or before the AF diagnosis date were extracted. AF and comorbidities were identified using medical codes in CPRD and International Classification of Diseases tenth revision codes in Hospital Episodes Statistic. Medication use was identified using product codes in CPRD and was defined as having at least 1 prescription recorded during the follow-up period. All used codes are listed in Supplemental Table 1.

### Study population

**Figure 1:**
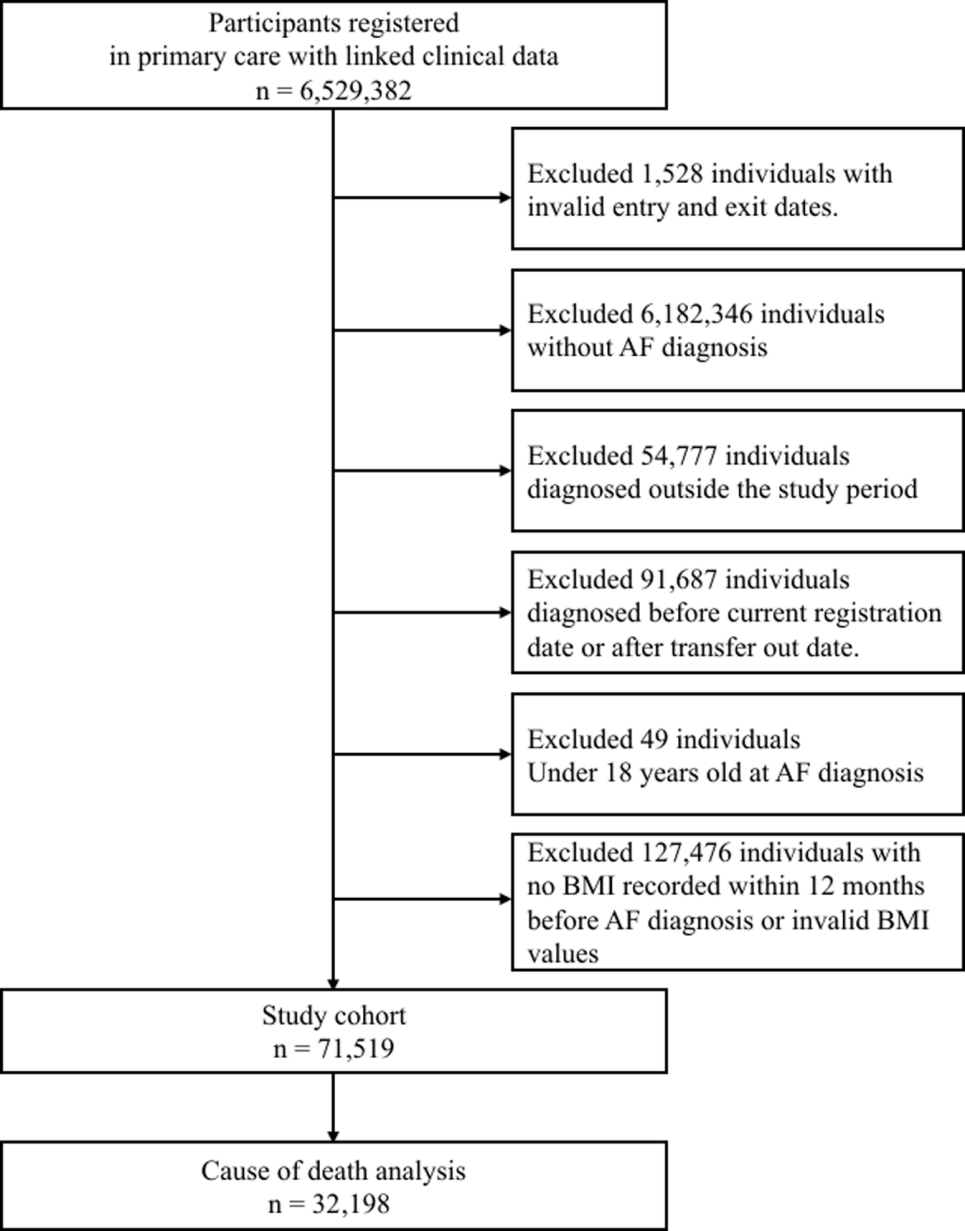
**Flowchart of the Study Population** AF = atrial fibrillation; BMI = body mass index.

Figure 1 illustrates the patient selection process and exclusion criteria. Individuals were excluded if they had a history of AF before study entry or were younger than 18 years at AF diagnosis. To ensure data quality, we also excluded individuals whose AF diagnosis occurred before their current registration date or after their transfer-out date. From the eligible AF cohort, individuals were included if they had a valid BMI measurement recorded within 12 months before their AF diagnosis. Extreme BMI values (<12 or >60 kg/m^2^) were excluded, as these were likely due to data entry errors or represented uncommon values that could bias the analysis.^17^

### Statistical analysis

Categorical variables were summarized as proportions (%), and continuous variables as mean ± SD or median (IQR), as appropriate. Unadjusted and multivariable Cox proportional hazards models were used to assess associations between BMI categories and the primary and secondary outcomes. Multivariable Cox proportional hazard models were adjusted for age, sex, ethnicity, deprivation, smoking, alcohol-related disorder, HF, hypertension, ischemic stroke, myocardial infarction, venous thromboembolism (VTE), peripheral artery disease (PAD), diabetes mellitus, chronic kidney disease (CKD), dyslipidemia, obstructive sleep apnea, chronic obstructive pulmonary disease (COPD), fatty liver, and asthma. Results are presented as HRs with 95% CIs. To allow for potential nonlinear associations, we modeled BMI using a restricted cubic spline with 4 knots. The reference BMI value was set at 22 kg/m^2^. The chi-square test was used to compare the differences in the proportions of specific causes of death among the 4 BMI groups. To estimate the incidence rate of ischemic stroke hospitalization according to OAC use, we selected patients with a follow-up duration of longer than 30 days.

Missing data were handled using multiple imputation by chained equations. Ten imputed data sets were generated, and estimates were pooled using Rubin rules. Complete-case analyses were also performed as a sensitivity analysis.

All analyses were conducted within the secure Data Safe Haven environment, compliant with data security and information governance requirements of University College London, National Health Service Digital, and Office for National Statistics, using R (version 4.3.0, R Foundation for Statistical Computing) and RStudio (version 1.2, Posit Software, PBC). Participant consent for data sharing was obtained. We followed the Strengthening the Reporting of Observational Studies in Epidemiology (STROBE) statement.^18^

### Ethic

The use of these data was approved by the Medicines and Healthcare Products Regulatory Agency Independent Scientific Advisory Committee (protocol 17_205).

## Results

### Cohort description

In this complete case analysis, 71,519 patients diagnosed with AF between 1998 and 2016 were included (Figure 1). Patients were categorized into 4 BMI groups: underweight (n = 2,472), normal weight (n = 21,731), overweight (n = 25,048), and obese (n = 22,268).

The mean age of the overall cohort was 74.9 ± 11.3 years, with a clear inverse relationship between age and BMI. Patients in the underweight group were the oldest (80.4 ± 10.6), whereas those in the obese group were the youngest (70.9 ± 10.9). Overall, 44.9% of the cohort was females. The proportion of women was highest in the underweight group (70.2%) and lowest in the overweight group (38.0%); the normal-weight and obese groups had similar proportions of women (49.6% and 45.2%, respectively) (Table 1).

**Table 1:** Baseline Population Characteristics

	Overall (N = 71,519)	Underweight (n = 2,472)	Normal (n = 21,731)	Overweight (n = 25,048)	Obese (n = 22,268)
Sex					
Men	39,413 (55.1%)	736 (29.8%)	10,950 (50.4%)	15,518 (62.0%)	12,209 (54.8%)
Women	32,106 (44.9%)	1,736 (70.2%)	10,781 (49.6%)	9,530 (38.0%)	10,059 (45.2%)
Age, y	74.9 (11.3)	80.4 (10.6)	78.0 (11.2)	75.0 (10.6)	70.9 (10.9)
Ethnicity					
Non-White	1,922 (2.8%)	67 (2.9%)	576 (2.8%)	670 (2.8%)	609 (2.8%)
White	66,668 (97.2%)	2,269 (97.1%)	20,122 (97.2%)	23,403 (97.2%)	20,874 (97.2%)
Most deprived quintile	17,236 (24.1%)	632 (25.6%)	5,072 (23.3%)	5,787 (23.1%)	5,745 (25.8%)
Smoking					
Nonsmoker	23,906 (33.8%)	766 (31.5%)	7,490 (34.9%)	8,278 (33.5%)	7,372 (33.4%)
Current or ex-smoker	46,789 (66.2%)	1,665 (68.5%)	14,002 (65.1%)	16,455 (66.5%)	14,667 (66.6%)
Alcohol-related disorder	2,682 (3.8%)	125 (5.1%)	751 (3.5%)	843 (3.4%)	963 (4.3%)
Heart failure	15,728 (22.0%)	538 (21.8%)	4,836 (22.3%)	5,186 (20.7%)	5,168 (23.2%)
Hypertension	50,064 (70.0%)	1,402 (56.7%)	13,769 (63.4%)	17,589 (70.2%)	17,304 (77.7%)
Ischemic stroke	4,630 (6.5%)	158 (6.4%)	1,508 (6.9%)	1,674 (6.7%)	1,290 (5.8%)
Myocardial infarction	13,157 (18.4%)	336 (13.6%)	3,966 (18.3%)	4,955 (19.8%)	3,900 (17.5%)
VTE	5,306 (7.4%)	141 (5.7%)	1,366 (6.3%)	1,752 (7.0%)	2,047 (9.2%)
PAD	6,676 (9.3%)	288 (11.7%)	2,117 (9.7%)	2,360 (9.4%)	1,911 (8.6%)
Diabetes mellitus	19,673 (27.5%)	284 (11.5%)	3,940 (18.1%)	6,838 (27.3%)	8,611 (38.7%)
CKD	737 (1.0%)	17 (0.7%)	240 (1.1%)	223 (0.9%)	257 (1.2%)
Dyslipidemia	23,821 (33.3%)	487 (19.7%)	6,014 (27.7%)	8,717 (34.8%)	8,603 (38.6%)
Obstructive sleep apnea	1,173 (1.6%)	3 (0.1%)	70 (0.3%)	211 (0.8%)	889 (4.0%)
COPD	34,413 (48.1%)	1,464 (59.2%)	10,148 (46.7%)	11,474 (45.8%)	11,327 (50.9%)
Fatty liver	393 (0.5%)	7 (0.3%)	78 (0.4%)	116 (0.5%)	192 (0.9%)
Asthma	12,716 (17.8%)	497 (20.1%)	3,543 (16.3%)	4,153 (16.6%)	4,523 (20.3%)
Warfarin or DOAC	32,762 (45.8%)	451 (18.2%)	7,812 (35.9%)	12,066 (48.2%)	12,433 (55.8%)
Warfarin	29,464 (41.2%)	409 (16.5%)	6,995 (32.2%)	10,910 (43.6%)	11,150 (50.1%)
DOAC	5,232 (7.3%)	65 (2.6%)	1,244 (5.7%)	1,828 (7.3%)	2,095 (9.4%)
Aspirin or clopidgrel	41,241 (57.7%)	1,071 (43.3%)	11,946 (55.0%)	15,071 (60.2%)	13,153 (59.1%)
Aspirin	53,425 (74.7%)	1,572 (63.6%)	15,828 (72.8%)	19,249 (76.8%)	16,776 (75.3%)
Clopidgrel	9,624 (13.5%)	238 (9.6%)	2,706 (12.5%)	3,652 (14.6%)	3,028 (13.6%)
Digoxin	21,863 (30.6%)	729 (29.5%)	6,657 (30.6%)	7,524 (30.0%)	6,953 (31.2%)

Values are n (%) or mean (SD).

CKD = chronic kidney disease; COPD = chronic obstructive pulmonary disease; DOAC = direct oral anticoagulant; PAD = peripheral artery disease; VTE = venous thromboembolism.

A slightly higher proportion of individuals in the most deprived socioeconomic quintile was observed in the underweight and obese groups (25.6% and 25.8%, respectively). Current or former smokers comprised 66.2% of the cohort, with a marginally higher prevalence in the underweight group (68.5%). Alcohol-related disorders were reported in 3.4% of patients overall and were most prevalent among obese individuals (4.5%).

Diabetes mellitus was substantially more common in overweight (27.3%) and obese patients (38.7%) compared with those of normal weight (18.1%) or underweight (11.5%). Similarly, hypertension prevalence increased across BMI categories, affecting 56.7% of underweight, 63.4% of normal-weight, 70.2% of overweight, and 77.7% of obese individuals. The prevalence of prior myocardial infarction was broadly similar across BMI categories, though slightly lower in underweight patients (13.6%) compared with normal-weight, overweight, and obese groups (18.3%, 19.8%, and 17.5%, respectively). Baseline HF was most common among obese patients (23.2%), with relatively small differences across other BMI groups.

Previous VTE and obstructive sleep apnea were more prevalent in obese individuals. Asthma was more frequent in underweight and obese patients, whereas COPD and PAD were more common among underweight patients.

Warfarin use increased progressively across BMI categories, from 16.5% in the underweight group to 50.1% in the obese group. A similar upward trend was observed for direct oral anticoagulants (underweight: 3.2%; obese: 11.3%). For aspirin, the prevalence of use was comparable among normal-weight (72.8%), overweight (76.8%), and obese (75.3%), but substantial lower in the underweight group (63.6%). Digoxin use remained consistent across all BMI categories.

### Associations between BMI categories and all-cause mortality

**Figure 2:**
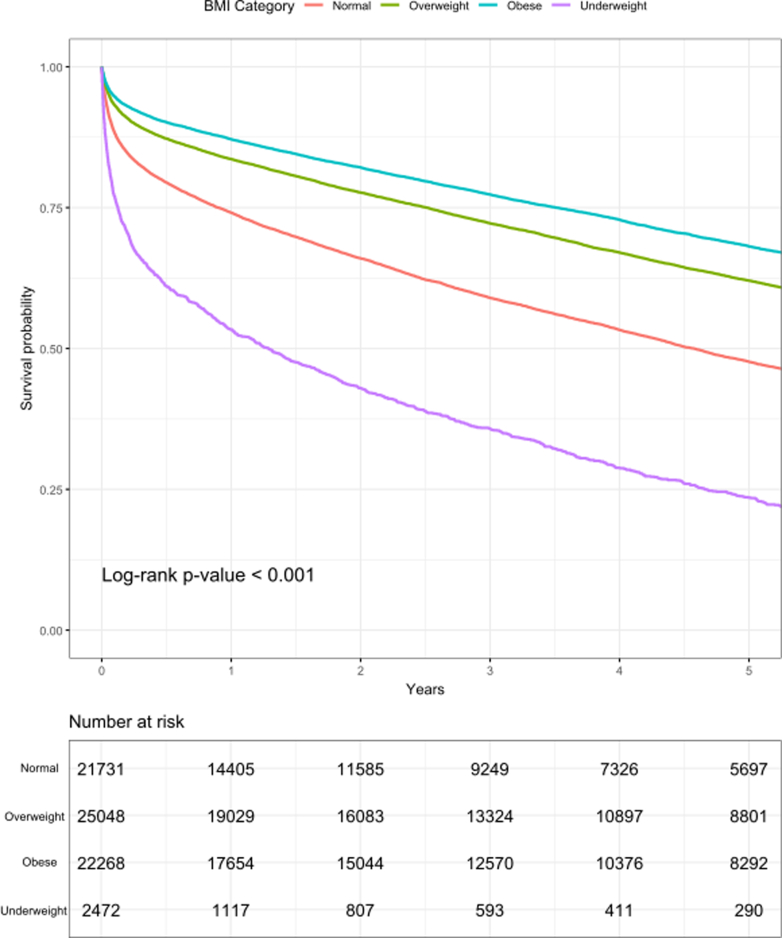
**Kaplan-Meier survival curves for All-Cause Mortality (5-Year Follow-Up)** Kaplan-Meier survival curves for all-cause mortality over a 5-year long-term follow-up period, stratified into 4 BMI categories: underweight, normal weight, overweight, and obesity. Abbreviation as in Figure 1.

**Figure 3:**
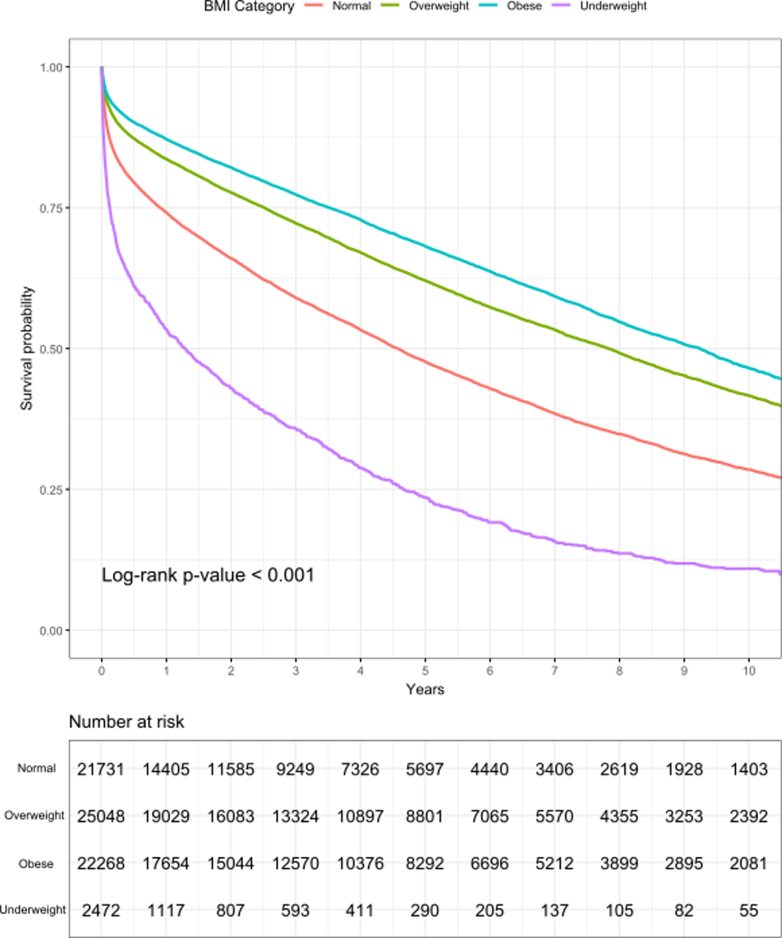
**Kaplan-Meier Survival Curves for All-Cause Mortality (Long Follow-Up)** Kaplan-Meier survival curves for all-cause mortality over a 10-year long-term follow-up period, stratified into 4 BMI categories: underweight, normal weight, overweight, and obesity. Abbreviation as in Figure 1.

Over a median follow-up of 5.72 years (IQR: 2.74-9.41), 32,198 patients (45.0%) died. Figures 2 and 3 show cumulative mortality across BMI categories at medium-term (5-year) and long-term (12-year) follow-up, with clear separation of survival curves that persisted beyond 10 years. The incidence ratio of all-cause death is 11.3 per 100 person-years.

In unadjusted Cox proportional hazards models, underweight patients had a significantly higher risk of all-cause mortality compared with normal-weight individuals (HR: 2.03; 95% CI: 1.93-2.13; *P* < 0.001). In contrast, overweight (HR: 0.66; 95% CI: 0.64-0.67; *P* < 0.001) and obese patients (HR: 0.55; 95% CI: 0.53-0.56; *P* < 0.001) had a significantly lower mortality risk. These associations remained significant after multivariable adjustment: underweight (HR: 1.87; 95% CI: 1.77-1.96; *P* < 0.001), overweight (HR: 0.72; 95% CI: 0.70-0.74; *P* < 0.001), and obese (HR: 0.69; 95% CI: 0.67-0.71; *P* < 0.001) (Table 2).

**Table 2:** Multivariate Predictors for All-Cause Mortality and Heart Failure Hospitalization

Variable	All-Cause Mortality			Heart Failure Hospitalization		
	HR	95% CI	*P* Value	HR	95% CI	*P* Value
Obese	0.69	0.67-0.71	<0.001	1.19	1.11-1.26	<0.001
Overweight	0.72	0.70-0.74	<0.001	0.94	0.88-1.00	0.04
Underweight	1.87	1.77-1.96	<0.001	0.91	0.76-1.09	0.29
Ref normal weight						
Age (per year)	1.06	1.06-1.06	<0.001	1.04	1.04-1.04	<0.001
Female	0.87	0.85-0.89	<0.001	0.89	0.85-0.94	<0.001
White ethnicity	0.99	0.92-1.07	0.83	0.67	0.59-0.76	<0.001
Most deprived quintile	1.11	1.08-1.13	<0.001	1.11	1.05-1.17	<0.001
Current or ex-smokers	1.21	1.18-1.25	<0.001	1.10	1.04-1.16	<0.001
Alcohol-related disorder	1.48	1.39-1.57	<0.001	1.06	0.92-1.22	0.41
Heart failure	1.69	1.65-1.74	<0.001	3.44	3.28-3.62	<0.001
Hypertension	1.01	0.98-1.03	0.50	1.13	1.07-1.20	<0.001
Ischemic stroke	1.33	1.28-1.39	<0.001	0.95	0.85-1.05	0.31
Myocardial infarction	1.18	1.15-1.21	<0.001	1.47	1.39-1.55	<0.001
VTE	1.23	1.19-1.28	<0.001	1.07	0.98-1.16	0.14
PAD	1.35	1.30-1.40	<0.001	1.24	1.16-1.33	<0.001
Diabetes mellitus	1.33	1.29-1.36	<0.001	1.38	1.31-1.46	<0.001
CKD	2.27	2.07-2.48	<0.001	0.98	0.77-1.25	0.88
Dyslipidemia	0.87	0.85-0.89	<0.001	0.95	0.90-1.00	0.06
Obstructive sleep apnea	1.02	0.92-1.14	0.68	1.25	1.06-1.49	0.009
COPD	1.18	1.16-1.21	<0.001	1.17	1.12-1.23	<0.001
Fatty liver	1.34	1.14-1.58	<0.001	1.10	0.79-1.54	0.57
Asthma	1.11	1.08-1.14	<0.001	1.09	1.03-1.16	0.006

Abbreviations as in Table 1.

Higher mortality risk was independently associated with increasing age (HR: 1.06; 95% CI: 1.06-1.06; *P* < 0.001), socioeconomic deprivation (HR: 1.11; 95% CI: 1.08-1.13; *P* < 0.001), current or ex-smokers (HR: 1.21; 95% CI: 1.18-1.24; *P* < 0.001), alcohol-related disorder (HR: 1.48; 95% CI: 1.39-1.57; *P* < 0.001), previous diagnosis of HF (HR: 1.69; 95% CI: 1.65-1.74; *P* < 0.001), previous ischemic stroke (HR: 1.33; 95% CI: 1.28-1.39; *P* < 0.001), previous myocardial infarction (HR: 1.18; 95% CI: 1.15-1.21; *P* < 0.001), VTE (HR: 1.23; 95% CI: 1.19-1.28; *P* < 0.001), PAD (HR: 1.35; 95% CI: 1.31-1.40; *P* < 0.001), diabetes mellitus (HR = 1.33; 95% CI: 1.29-1.36; *P* < 0.001), CKD (HR: 2.27; 95% CI: 2.07-2.48; *P* < 0.001), COPD (HR: 1.18; 95% CI: 1.16-1.21; *P* < 0.001), fatty liver (HR: 1.34; 95% CI: 1.14-1.58; *P* < 0.001), and asthma (HR: 1.11; 95% CI: 1.08-1.14; *P* < 0.001). Female sex (HR: 0.87; 95% CI: 0.85-0.89; *P* < 0.001) and dyslipidaemia (HR: 0.87; 95% CI: 0.85-0.89; *P* < 0.001) were significantly associated with a lower mortality risk.

The sensitivity analysis using complete case analysis showed nearly identical HRs and a consistent trend to the primary analysis (Supplemental Table 2).

### Associations between BMI categories and other cardiovascular outcomes

**Figure 4:**
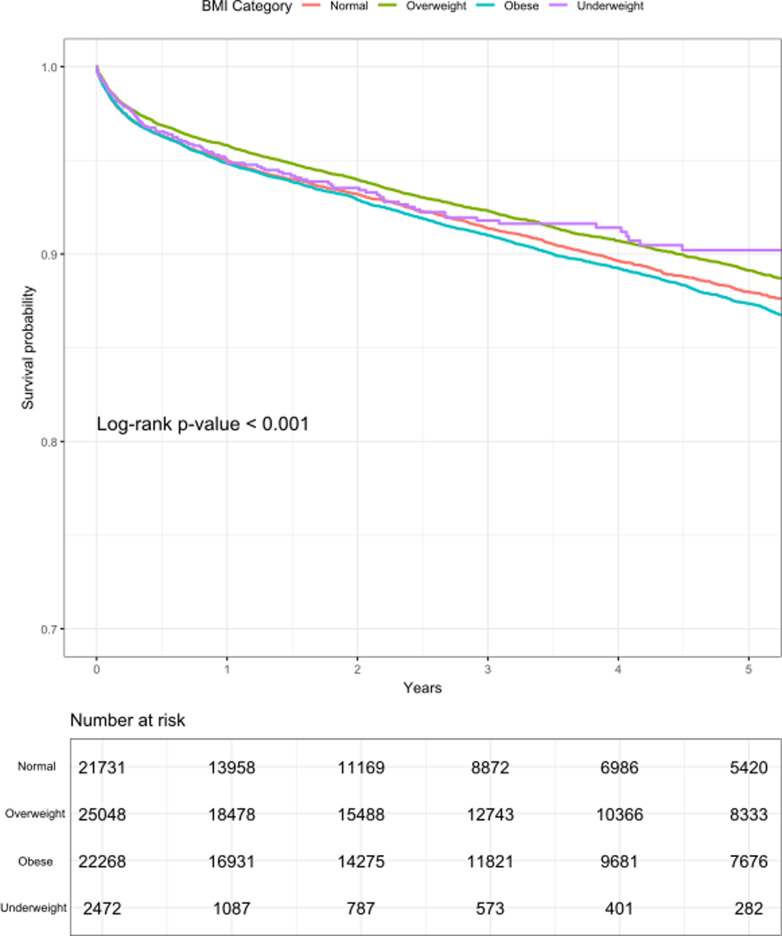
**Kaplan-Meier Survival Curves for Heart Failure Hospitalization (5-Year Follow-Up)** Kaplan-Meier survival curves for heart failure hospitalization over a 5-year long-term follow-up period, stratified into 4 BMI categories: underweight, normal weight, overweight, and obesity. Abbreviation as in Figure 1.

During follow-up, 7,152 patients (10.0%) were hospitalized for new or worsening HF (Figure 4, Supplemental Figure 1). The incidence rate of HF hospitalization was 2.64 per 100 person-years. In unadjusted analyses, obese patients had a higher risk of HF hospitalization compared with normal-weight individuals (HR: 1.12; 95% CI: 1.05-1.19; *P* < 0.001), whereas overweight patients had a lower risk (HR: 0.92; 95% CI: 0.86-0.97; *P* < 0.001). After adjustment, obesity remained associated with an increased risk (HR: 1.19; 95% CI: 1.11-1.26; *P* < 0.001), whereas overweight status was associated with a modestly reduced risk (HR: 0.94; 95% CI: 0.88-1.00; *P* = 0.04).

Higher risk of heart-failure hospitalization was also associated with older age, socioeconomic deprivation, smoking, prior HF, hypertension, previous myocardial infarction, PAD, diabetes mellitus, obstructive sleep apnea, and COPD (Table 2). Female sex (HR: 0.89; 95% CI: 0.85-0.94; *P* < 0.001) and White ethnicity (HR: 0.67; 95% CI: 0.59-0.76; *P* < 0.001) were associated with lower risk.

Hospitalization for myocardial infarction occurred in 3,237 patients (4.53%) during follow-up (Supplemental Figures 2 and 3). The incidence rate of myocardial infarction hospitalization was 1.17 per 100 person-years. Obesity was associated with a lower risk of myocardial infarction hospitalization in both unadjusted (HR: 0.86; 95% CI: 0.79-0.94; *P* < 0.001) and adjusted analyses (HR: 0.91; 95% CI: 0.82-1.00; *P* = 0.04) (Table 3). Increased risk was associated with older age, smoking, prior HF, hypertension, prior myocardial infarction, PAD, diabetes mellitus, CKD, dyslipidaemia, fatty liver, and asthma.

**Table 3:** Multivariate Predictors for Myocardial Infarction Hospitalization and Ischemic Stroke Hospitalization

Variable	Myocardial Infarction Hospitalization			Ischemic Stroke Hospitalization			Bleeding Hospitalization		
	HR	95% CI	*P* Value	HR	95% CI	*P* Value	HR	95% CI	*P* Value
Obese	0.91	0.82-1.00	0.04	0.95	0.87-1.05	0.32	0.87	0.81-0.92	<0.001
Overweight	0.94	0.86-1.03	0.16	1.06	0.97-1.14	0.20	0.92	0.87-0.97	0.003
Underweight	1.24	0.98-1.55	0.07	1.06	0.85-1.31	0.63	0.88	0.74-1.05	0.15
Ref normal weight									
Age (per year)	1.04	1.03-1.04	<0.001	1.05	1.05-1.06	<0.001	1.02	1.02-1.02	<0.001
Female	0.95	0.88-1.03	0.20	1.35	1.26-1.45	<0.001	0.81	0.77-0.85	<0.001
White ethnicity	0.85	0.69-1.04	0.10	0.78	0.64-0.96	0.01	1.01	0.88-1.17	0.85
Most deprived quintile	1.06	0.98-1.15	0.12	1.03	0.95-1.11	0.43	0.95	0.90-100	0.047
Current or ex-smokers	1.15	1.07-1.25	<0.001	1.04	0.97-1.11	0.30	1.01	0.96-1.06	0.70
Alcohol-related disorder	1.05	0.85-1.29	0.64	1.26	1.02-1.54	0.03	1.40	1.25-1.57	<0.001
Heart failure	1.30	1.20-1.41	<0.001	0.95	0.87-1.04	0.30	1.14	1.07-1.2	<0.001
Hypertension	1.26	1.15-1.37	<0.001	1.27	1.17-1.37	<0.001	1.22	1.16-1.29	<0.001
Ischemic stroke	1.00	0.87-1.16	0.98	3.25	2.96-3.56	<0.001	1.13	1.03-1.24	0.01
Myocardial infarction	2.88	2.67-3.11	<0.001	1.05	0.96-1.15	0.31	1.00	0.94-1.06	0.99
VTE	1.06	0.93-1.21	0.39	0.91	0.80-1.04	0.18	1.13	1.04-1.23	0.005
PAD	1.41	1.28-1.56	<0.001	1.14	1.01-1.28	0.03	1.10	1.02-1.19	0.01
Diabetes mellitus	1.46	1.36-1.58	<0.001	1.25	1.16-1.35	<0.001	1.12	1.07-1.18	<0.001
CKD	2.04	1.57-2.66	<0.001	1.01	0.66-1.54	0.95	1.89	1.54-2.32	<0.001
Dyslipidemia	1.09	1.01-1.18	0.02	1.01	0.94-1.09	0.72	1.03	0.98-1.08	0.23
Obstructive sleep apnea	0.81	0.59-1.13	0.22	0.81	0.56-1.19	0.29	1.30	1.10-1.54	0.002
COPD	1.08	1.00-1.16	0.05	0.96	0.89-1.02	0.20	1.07	1.02-1.13	0.003
Fatty liver	1.71	1.12-2.59	0.01	1.23	0.71-2.13	0.46	1.57	1.19-2.09	0.002
Asthma	1.14	1.04-1.26	0.004	0.86	0.78-0.95	0.003	1.09	1.03-1.16	0.004

Abbreviations as in Table 1.

During follow-up, 3,604 patients (5.04%) were hospitalized for ischemic stroke (Supplemental Figures 4 and 5). The incidence rate of ischemic stroke hospitalization was 1.30 per 100 person-years. Although obese patients had a lower risk in unadjusted analyses (HR: 0.75; 95% CI: 0.69-0.82; *P* < 0.001), this association was no longer significant after adjustment (HR: 0.95; 95% CI: 0.87-1.05; *P* = 0.32). Higher stroke risk was independently associated with older age, female sex, alcohol-related disorders, hypertension, prior ischemic stroke, PAD, and diabetes mellitus. White ethnicity (HR: 0.78; 95% CI: 0.64-0.96) and asthma (HR: 0.86; 95% CI: 0.78-0.95) were associated with a lower stroke risk (Table 3). The incidence rates of ischemic stroke hospitalization without and with OAC use were 1.29 vs 1.02 per 100 person-years, respectively.

During follow-up, 7,900 patients (11.0%) were hospitalized for bleeding (Supplemental Figures 6 and 7). The incidence rate of bleeding hospitalization was 3.01 per 100 person-years. In the unadjusted analysis, both obesity and overweight were associated with a lower risk (HR: 0.87; 95% CI: 0.81-0.92; *P* < 0.001 and HR: 0.92; 95% CI: 0.87-0.98; *P* = 0.005, respectively). These associations remained consistent in the adjusted model for obesity (HR: 0.87; 95% CI: 0.81-0.92; *P* < 0.001) and for overweight (HR: 0.92; 95% CI: 0.87-0.97; *P* = 0.003).

Higher bleeding risk was associated with older age, alcohol-related disorders, HF, hypertension, prior ischemic stroke, VTE, PAD, diabetes mellitus, CKD, obstructive sleep apnea, COPD, fatty liver, and asthma. Female sex and socioeconomic deprivation were associated with a lower bleeding risk (Table 3).

The complete case analysis for cardiovascular outcomes showed nearly identical hazard ratios and a consistent trend to the primary analysis (Supplemental Table 2).

### Nonlinear associations between continuous BMI and clinical

**Figure 5:**
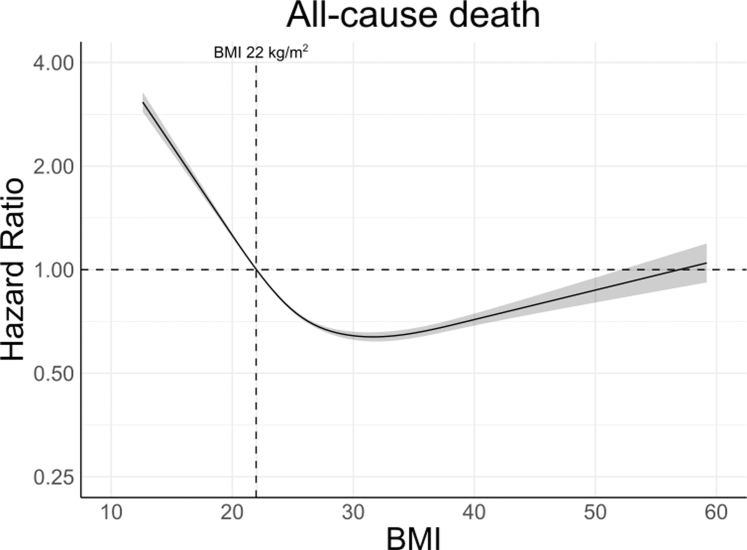
**Spline Curve for All-Cause Mortality** Spline curves for all-cause mortality, showing HRs on the y-axis and BMI on the x-axis. The vertical black dotted line indicates the reference BMI of 22 kg/m^2^, and the horizontal black dotted line represents a hazard ratio of 1.0. Abbreviation as in Figure 1.

**Figure 6:**
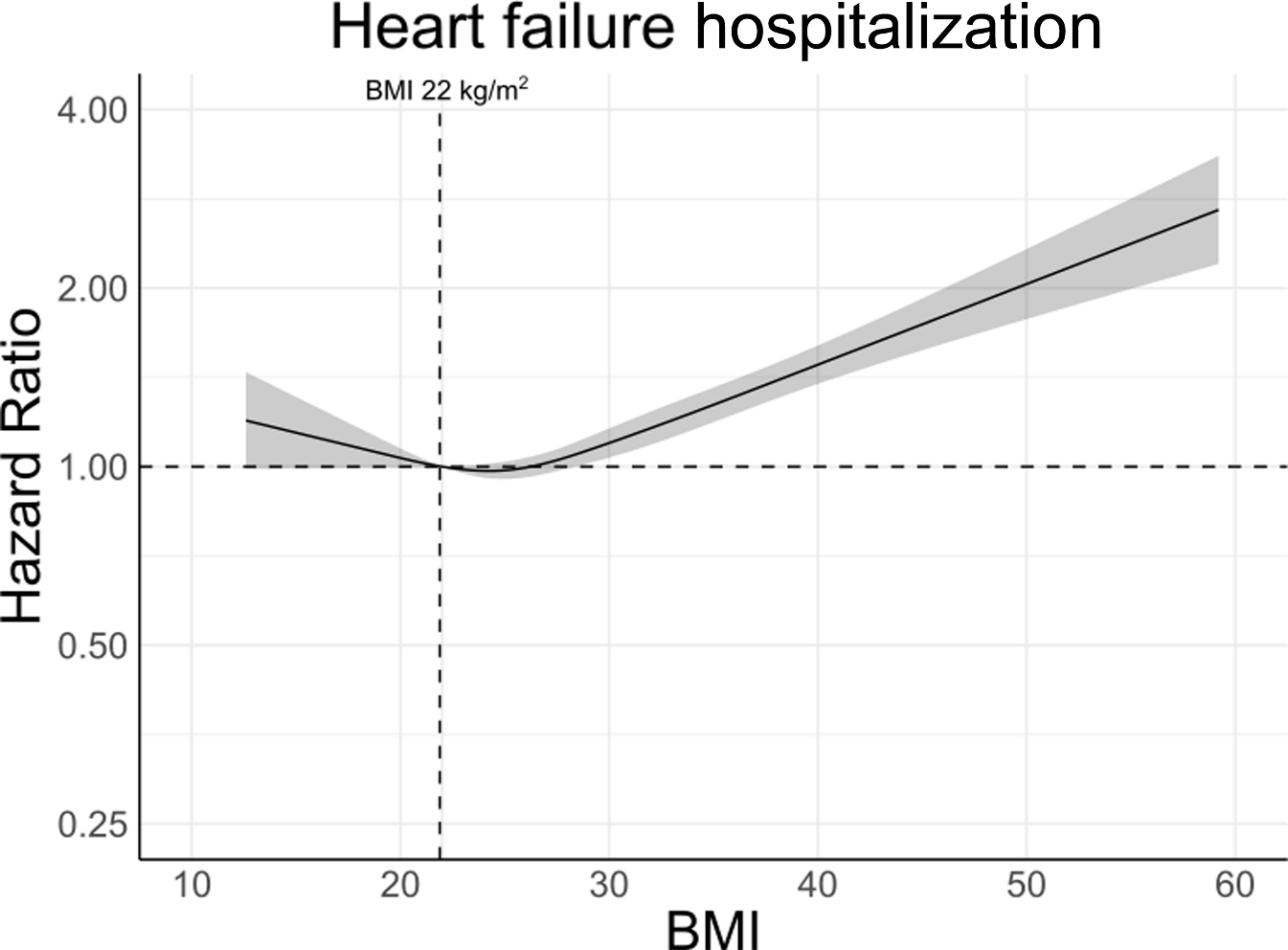
**Spline Curves for Heart Failure Hospitalization** Spline curves for heart failure hospitalization, showing HRs on the y-axis and BMI on the x-axis. The vertical black dotted line indicates the reference BMI of 22 kg/m^2^, and the horizontal black dotted line represents a hazard ratio of 1.0. Abbreviation as in Figure 1.

For all-cause mortality and HF hospitalization, U-shape associations were observed (both nonlinearity *P* value <0.001) (Figures 5 and 6). The risk of all-cause mortality was lowest at a BMI of approximately 30 kg/m^2^. The HR was substantially higher in the underweight range compared to the obese range. For HF hospitalization, the lowest HR was observed for approximately 22 kg/m^2^. Although the risk was slightly higher in underweight individuals, it showed a gradual increase across the overweight and obese. In contrast, the association for ischemic stroke hospitalization and myocardial infarction hospitalization showed a largely linear pattern (nonlinearity *P* value = 0.12 and 0.92, respectively) (Supplemental Figures 8 and 9). Higher BMI showed marginally lower risk for myocardial infarction, whereas for ischemic stroke hospitalization, higher BMI was associated with a lower risk of ischemic stroke hospitalization. Although the risk of bleeding remained stable below a BMI of 22 kg/m^2^, it gradually decreased above this threshold, reaching its lowest level at approximately 30 to 35 kg/m^2^. Thereafter, the risk showed a progressive increase.

### Causes of death across BMI categories

Across all BMI groups, disease of the circulatory system, neoplasms and diseases of the respiratory system were the 3 most common causes of death (Table 4). Diseases of the circulatory system were the leading cause of death in the normal, overweight, and obese groups, accounting for 38.0%, 41.6%, and 43.4% of deaths, respectively (*P* < 0.001). In contrast, for underweight patients, disease of the respiratory system were the most common cause of death (31.3%). Deaths due to neoplasms were most frequent in the overweight group (22.7%).

**Table 4:** Cause of Death Analysis by Weight Category

	Overall	Underweight	Normal	Overweight	Obese	*P* Value
Diseases of the circulatory system	12,896 (40.1)	510 (28.9)	4,455 (38.0)	4,410 (41.6)	3,521 (43.4)	<0.001
Neoplasms	6,880 (21.4)	269 (15.3)	2,423 (20.7)	2,403 (22.7)	1785 (22.0)	<0.001
Diseases of the respiratory system	5,265 (16.4)	551 (31.3)	2,111 (18.0)	1,539 (14.5)	1,064 (13.1)	<0.001
Diseases of the digestive system	1,508 (4.7)	73 (4.1)	538 (4.6)	463 (4.4)	434 (5.4)	0.01
Mental and behavioral disorders	926 (2.9)	74 (4.2)	426 (3.6)	268 (2.5)	158 (2.0)	<0.001
Diseases of the genitourinary system	762 (2.4)	28 (1.6)	278 (2.4)	257 (2.4)	199 (2.5)	0.17
Endocrine, nutritional, and metabolic diseases	701 (2.2)	21 (1.2)	162 (1.4)	230 (2.2)	288 (3.6)	<0.001
External causes of morbidity and mortality	576 (1.8)	30 (1.7)	244 (2.1)	196 (1.9)	106 (1.3)	<0.001
Diseases of the nervous system	492 (1.5)	42 (2.4)	211 (1.8)	160 (1.5)	79 (1.0)	<0.001
Certain infectious and parasitic diseases	404 (1.3)	30 (1.7)	151 (1.3)	126 (1.2)	97 (1.2)	0.31
Symptoms, signs and abnormal clinical and laboratory findings, not elsewhere classified	294 (0.9)	23 (1.3)	150 (1.3)	74 (0.7)	47 (0.6)	<0.001
Diseases of the musculoskeletal system and connective tissue	249 (0.8)	26 (1.5)	99 (0.8)	58 (0.6)	66 (0.8)	<0.001
Diseases of the skin and subcutaneous tissue	169 (0.5)	8 (0.5)	40 (0.3)	53 (0.5)	68 (0.8)	<0.001
Diseases of the blood and blood-forming organs and certain disorders involving the immune mechanism	54 (0.2)	5 (0.3)	22 (0.2)	18 (0.2)	9 (0.1)	0.35
Congenital malformations, deformations and chromosomal abnormalities	25 (0.1)	2 (0.1)	13 (0.1)	5 (0.05)	5 (0.1)	0.32
Codes for special purposes	5 (0.02)	0 (0)	1 (0.01)	1 (0.01)	3 (0.04)	0.35
Diseases of the ear and mastoid process	2 (0.01)	0 (0)	0 (0)	1 (0.01)	1 (0.01)	0.68
Diseases of the eye and adnexa	1 (0.003)	0 (0)	0 (0)	1 (0.01)	0 (0)	0.56
Recorded in ICD-9	827 (2.6)	60 (3.4)	356 (3.0)	283 (2.7)	128 (1.6)	<0.001
No record	162 (0.5)	10 (0.56)	41 (0.4)	55 (0.5)	56 (0.7)	0.01

ICD = International Classification of Diseases ninth revision.

## Discussion

In this study our principal findings are as follows: 1) most of the AF population in the United Kingdom can be classified as either obese or overweight. Obese and overweight AF patients were predominantly men and demonstrated a higher prevalence of established cardiovascular risk factors, whereas underweight AF patients were more frequently women, smokers or ex-smokers, reported more alcohol-related issues, with lower rates of hypertension, diabetes, and dyslipidemia; 2) overweight and obese individuals demonstrated lower all-cause mortality compared with those of normal weight, even after adjustment for baseline confounders, whereas underweight individuals experienced higher mortality rates; 3) obesity was independently associated with an increased risk of HF hospitalization and lower rates of hospitalization for myocardial infarction and bleeding; and 4) we show novel data on cause-specific mortality per BMI class within the AF population, providing a rationale for the differences observed across the groups (Central Illustration).

**Central Illustration:**
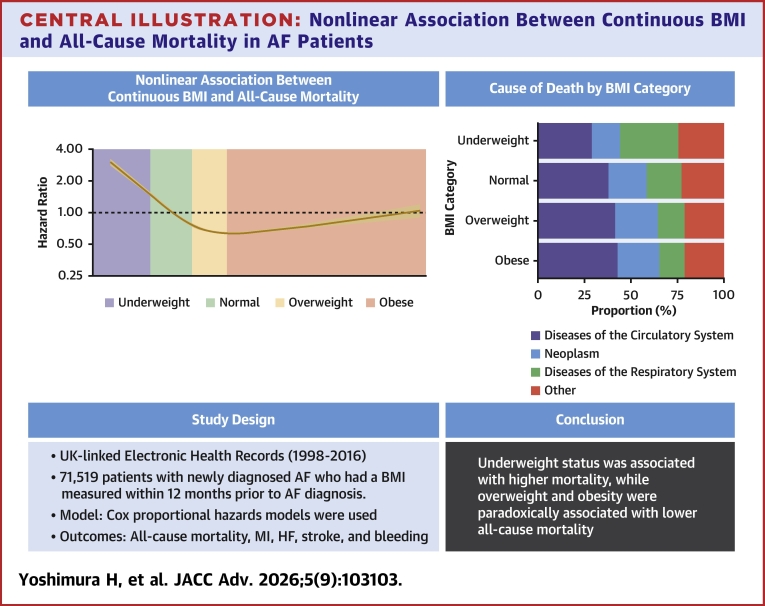
**Nonlinear Association Between Continuous BMI and All-Cause Mortality in AF Patients** AF = atrial fibrillation; BMI = body mass index; HF = heart failure; MI = myocardial infarction.

Observational registry data support our finding that nearly 40% of patients with AF are overweight and about one-fifth are obese.^19^, ^20^, ^21^ The GARFIELD-AF registry, which followed >52,000 newly diagnosed AF patients for 2 years, similarly observed an increased risk of new or worsening HF in obese individuals after adjustment, as well as a higher mortality risk in both underweight and obese patients.^19^ Consistent with our results, the ORBIT-AF reported that higher BMI was associated with lower adjusted risk of all-cause mortality.^20^ The smaller EORP-AF registry suggested lower all-cause mortality in obese women with AF followed for 1 year, but this association did not persist after adjustment.^21^ Rigutini et al^22^ have suggested that clinical complexity (defined as a CHA_2_DS_2_-VASc ≥2 and the presence of at least 1 of the following: elderly age, low BMI, CKD, or a history of major bleeding) in the AF population increases the risk of major adverse outcomes, such as all-cause death, major adverse cardiovascular events, and major bleeding. As all our underweight patients fall into this definition of clinical complexity, the observed high mortality aligns with the findings by Rigutini et al,^22^ confirming that clinical complexity is a key determinant of poor outcomes.

The stronger signal for increased HF hospitalizations among obese patients may reflect shared genetic correlations between AF, HF, and obesity.^23^ The higher mortality seen in underweight individuals requires further investigation; proposed mechanisms include malnutrition, reduced physiological reserve, immunosenescence, sarcopenia, and frailty.^24^ Reverse causation is also possible, as being underweight can signal underlying conditions such as cancer, dementia, or end-stage organ failure with cachexia. The high mortality observed in the first year after AF diagnosis in underweight patients likely indicates that within this class there is possibly a subgroup of patients who have been losing weight due to severe comorbidities and that were classified as underweight at the time of AF diagnosis. It is plausible that the progression of these underlying comorbidities, led to issues in multiple organ systems, triggered weight loss, and eventually led to AF shortly before death. We hypothesize that it is this group of patients, with a descending weight trajectory, that drives increased mortality in this weight class during the first year as a result of respiratory, digestive, mental, musculoskeletal disorders and other conditions. On the other hand, another group of individuals with low BMI at the time of AF diagnosis, who likely had a low BMI for a while during their lives, present with a more stable health trajectory, and account for the events observed following the initial period that may be occurring as a result of aging or acute disease that develops long after their AF diagnosis. Optimizing care of underlying conditions driving a descending BMI trajectory around the time of new-onset AF could be a crucial strategy for improving the outcome of this patient group. Interventions should address both underweight and obese AF patients. Targeting weight loss in obese individuals may be of interest to reduce HF hospitalizations, whereas for overweight patients, emphasis should be on optimal control of traditional cardiovascular risk factors (hypertension, dyslipidemia, and diabetes)—often achievable with modest weight loss—to lower event risk.

Managing bleeding risk is a major determinant of the continuation of OAC therapy, as bleeding events frequently lead to treatment interruption and nonadherence to therapy, and a subsequent increase in risk of stroke and mortality.^25^ Our study showed no significant risk increase in bleeding for underweight patients, while observing a decreased risk for overweight and obese. Although a meta-analysis of 4 randomized clinical trials reported a gradually lower risk of bleeding for patients with a BMI over 20 kg/m^2^ on warfarin, the ETNA-AF registry indicated that a low BMI was associated with higher bleeding risks for AF patients on direct oral anticoagulants.^10^^,^^26^ In our data set, however, not all patients were prescribed OACs, and there was a notably low proportion of OAC and antiplatelet therapy among underweight patients, which may partially explain why we failed to observed an increase in bleeding risk in this patient group. Notably, obesity management is already recommended in global guidelines as part of holistic or integrated care management pathways.^27^^,^^28^ A large Swedish multicenter, prospective, matched controlled study suggested that weight loss achieved through bariatric surgery reduces the risk of new onset AF among individuals with morbid obesity.^29^ Also, weight reduction can reduce AF burden: for example, the REVERSE-AF study found lowered AF burden after weight loss in overweight/obese patients,^30^ and a systematic review showed weight loss reduces AF recurrence after catheter ablation.^31^ Recent therapies such as the glucagon-like peptide-1 receptor agonists (GLP-1 RAs)have shown potential benefits for improving outcomes in patients with obesity, with benefits seemingly independent of baseline adiposity and weight loss.^32^ A meta-analysis of 24 randomized controlled trials (40,694 participants) found GLP-1 RAs were associated with an 18% relative reduction in AF risk among overweight/obese individuals.^33^ The long-term outcomes of the LEAF (Liraglutide Effect on Atrial Fibrillation) trial evaluated risk factor modification with or without preablation liraglutide in patients with AF and a BMI >27 kg/m^2^. Although weight loss differences between groups were numerical but not statistically significant (−2.7 ± 5.7 vs −4.5 ± 6.5 kg), the liraglutide group demonstrated a significantly higher rate of freedom from AF recurrence off antiarrhythmic drugs at 3 years (67.5% vs 29.6%; *P* < 0.001).^34^ Further ongoing trials will clarify the role of this drug class in AF management.^35^^,^^36^ GLP-1 receptors are expressed in epicardial adipose tissue (EAT), where their activation by agonists is thought to reduce local adipogenesis, enhance fat utilization, and decrease EAT thickness. Additionally, these agents may induce brown fat differentiation and modulate the renin-angiotensin-aldosterone system.^37^ Evidence suggests that GLP-1RAs may modulate EAT inflammation, potentially prevent or even reverse fibrosis and autonomic dysregulation.^37^^,^^38^

A previous systematic review identified an obesity paradox for all-cause and cardiovascular mortality among AF patients across various ethnicities.^39^ Although it incorporated data from 13 studies (both randomized controlled trials and observational), the observational sample size was less than a third of our own; consequently, it was underpowered for several of the assessed outcomes. Several hypotheses have been proposed to explain this paradox.^39^ First, the relationship between lean and fat mass may be critical, as the lean mass index, rather than BMI alone, may drive the risk of adverse outcomes. Second, obese individuals tend to be younger as observed in our cohort. However, the association between high BMI and favorable outcomes persisted after adjusting for age. Finally, although shorter follow-up has been cited as a potential cause of the paradox, our study—which included over 20,000 individuals followed for 5 years or more—could not support this theory. Our cause-of-death analyses help explain the excess mortality in underweight patients: they had significantly higher deaths from circulatory and respiratory diseases compared with other BMI categories. Paradoxically, obese patients had lower mortality from circulatory diseases and neoplasms. Neoplasm was a major cause of death in our overall cohort. A previous analysis of this data set has shown that smoking, excessive alcohol intake, CKD, socioeconomic deprivation and other risk factors for cardiovascular disease are also associated with cancer in the AF population.^40^ In addition, shared risk factors and mechanisms between cardiovascular disease and cancer are reported, such as hypertension, hyperlipidemia, diabetes mellitus, obesity, smoking, physical inactivity, and the social determinants of health.^41^ Even though causation cannot be inferred for some of these, it is possible that an integrated management of AF patients with comprehensive treatment of correctable risk factors and comorbidities may impact patient on patients overall wellbeing, with the benefit extending beyond with cardiovascular health.

### Strengths and limitations

Strengths of our study include being the first nationwide analysis, the largest sample (over 5 times GARFIELD-AF and twenty times ORBIT-AF), sufficient power to examine specific cardiovascular outcomes (rather than composites), assessment of causes of death, and long follow-up (5,931 patients followed ≥10 years).

Some limitations need to be highlighted. First, as with any observational study, we report associations and cannot infer causality. Results may reflect survival bias, competing risks, or detection bias, as obese patients likely have more frequent health care contact that could influence outcome ascertainment (eg dyslipidemia was significantly associated with a lower risk for some of the outcomes which was likely due to the use of statins, and VTE was associated with lower risk of ischemic stroke, likely due to the use of anticoagulants). Second, we cannot exclude the possibility of unmeasured confounding. Third, the analysis relied on diagnostic coding and using BMI. Alternative measures such as waist-to-height ratio^42^ or Dual-Energy X-ray Absorptiometry^43^ may better capture adiposity. Fourth, we have not evaluated the impact of interval changes in BMI. Previous analyses of the CPRD data set show that younger adults are more likely to gain weight over a 10-year follow-up period, whereas >50% of older adults maintain stable weight.^44^ Pronounced weight loss in elderly individuals is usually unintentional, and associated with underlying severe disease, and linked to increased mortality.^45^ Finally, our study population was largely white British, and additional studies in non-White populations are needed, given the reported racial differences in clinical epidemiology^46^ and AF-related outcomes.^47^^,^^48^

## Conclusions

In this large, nationwide AF cohort, underweight status was associated with higher mortality, whereas overweight and obesity were paradoxically associated with lower all-cause mortality despite increased HF hospitalizations in obese patients. The rate of cardiovascular complications varies across the different BMI categories.

### Declaration of Generative AI and AI-Assisted Technologies in the Writing Process

During the preparation of this work the authors used Google Gemini and ChatGPT in order to check spell and grammar. After using this tool/service, the authors reviewed and edited the content as needed and take full responsibility for the content of the published article.

Perspectives **COMPETENCY IN MEDICAL KNOWLEDGE:** Our study demonstrated that patient backgrounds and the distribution of cardiovascular risk factors varied significantly across BMI categories. We found that obese individuals had a lower mortality rate, whereas underweight individuals experienced a higher mortality rate. Regarding cardiovascular events, obese patients had a higher incidence of hospitalisation for heart failure, but a lower incidence of hospitalisation for myocardial infarction. Furthermore, our analysis of cause-specific mortality showed that the distribution of causes of death varied according to BMI category.

**TRANSLATIONAL OUTLOOK:** Obesity management is already recommended in global guidelines as a core component of holistic and integrated care pathways. Independent of general obesity management, our findings highlight the necessity of considering more personalized treatment strategies based on BMI categories. Optimising AF management in this manner may help clinicians improve patient outcomes in an aging society and inform the allocation of healthcare resources and prevention efforts.

## Funding support and author disclosures

Dr Providencia is supported by 10.13039/501100000274British Heart Foundation (grant AA/18/6/34223), the 10.13039/501100000272National Institute for Health and Care Research (grant NIHR129463), and United Kingdom Research and Innovation (grant 10103153 ARISTOTELES). Dr Lip reports institutional consultancy and/or speaker roles for BMS/Pfizer, Boehringer Ingelheim, Anthos, Huawei (no fees are received personally). All other authors have reported that they have no relationships relevant to the contents of this paper to disclose.
