# Supplementary material for: Obesity Subphenotypes in Atrial Fibrillation: A Linked Electronic Health Record Study in the United Kingdom
Source: JACC Adv. 2026 Aug 5;5(9):103103. doi: 10.1016/j.jacadv.2026.103103 (PMC13474635; doi:10.1016/j.jacadv.2026.103103)

Supplementary Material

Table S.1. Utilized Electronic Health Record (EHR) codes

	**CPRD Medical codes**	**HES ICD-10 codes**
**Alcohol related disorder**	1399, 1476, 2081, 2082, 2083, 2084, 2925, 3216, 4500, 4501, 4506, 4743, 4915, 5611, 5740, 5758, 6169, 6467, 7123, 7602, 7885, 7943, 8030, 8363, 8388, 8430, 9489, 9849, 10691, 11106, 11107, 11670, 11740, 12353, 12442, 16225, 16237, 16587, 17259, 17330, 17607, 18156, 18636, 19494, 20514, 20762, 21624, 21650, 21713, 21879, 22277, 24064, 24485, 24984, 25110, 26106, 26323, 27342, 28780, 29691, 30162, 30404, 30460, 30604, 30695, 31443, 31742, 32927, 32964, 33635, 33670, 33839, 36296, 36748, 37605, 37691, 37946, 38061, 39327, 39799, 40530, 41920, 41983, 43193, 45169, 46677, 47555, 54505, 56410, 56947, 57714, 59574, 61383, 62000, 63529, 64101, 64389, 65754, 65932, 67651, 68111, 69691, 94553, 94670, 95181, 96053, 96054, 96993, 97309, 104611	E24.4, F10.1, F10.2, F10.3, F10.4, F10.5, F10.6, F10.7, F10.8, F10.9, G31.2, G62.1, G72.1, I42.6, K29.2, K70, K85.2, K86.0, Z50.2, Z71.4
**Asthma**	78, 185, 232, 233, 719, 1208, 1555, 1780, 2290, 3665, 4442, 4606, 4892, 5267, 5627, 5798, 5867, 6707, 7146, 7731, 8335, 10996, 11370, 11839, 12987, 14777, 15248, 16070, 18207, 18323, 21232, 22752, 25796, 27926, 29325, 32727, 40823, 41017, 45073, 45782, 58196, 73522, 106805, 2126200	J45, J46
**Atrial Fibrillation**	1268, 1664, 1757, 2212, 3272, 3273, 3757, 6771, 18746, 23437, 35127, 39114, 45773, 57832, 63350, 90187, 90188, 90189, 90190, 90191, 96076, 96277	I48
**Bleeding**	-	D62, D683, D698, D699, H113, H210, H313, H356, H431, H450, H922, I230, I312, I600, I601, I602, I603, I604, I605, I606, I607, I608, I609, I610, I611, I612, I613, I614, I615, I616, I618, I619, I620, I621, I629, I850, I983, J942, K226, K250, K252, K254, K256, K260, K262, K264, K266, K270, K272, K274, K276, K280, K282, K284, K286, K290, K625, K661, K762, K920, K921, K922, M250, N020, N021, N022, N023, N024, N025, N026, N027, N028, N029, N421, N920, N921, N923, N924, N930, N938, N939,

		N950, R040, R041, R042, R048, R049, R31, R58, S064, S065, S066, S260, S271, T792
**CKD**	2994, 2996, 2997, 5504, 5911, 6712, 8037, 8330, 11553, 11745, 11773, 12585, 18774, 20073, 20196, 22252, 23773, 24361, 26862, 28158, 30709, 30756, 36442, 44422, 45160, 46145, 46438, 48057, 48639, 49028, 52123, 53852, 54990, 59315, 60446, 60498, 60743, 63038, 63488, 63502, 64828, 66705, 66714, 69266, 69760, 70874, 72004, 72336, 74905, 86419, 88597, 89924, 93366, 95405, 95508, 96133, 96184, 97683, 98364, 98888, 99692, 100693, 101124, 101736, 101756, 101912, 103429, 104049, 104050, 104201, 104586, 104630, 104719, 104905, 104960, 105328, 105436, 105724, 105742, 105760, 105811, 106301, 106620, 106720, 106866, 106975, 107000, 107082, 107188, 107220, 107260, 107746, 107752, 107900, 108116, 108213, 108423, 108437, 108699, 108759, 108785, 109135, 109455, 109809, 110051, 110072, 110095	N16.5, N18.5, T82.4, T86.1, Y60.2, Y61.2, Y84.1, Z49.1, Z49.2, Z94.0, Z99.2, L74.6, M01.1, M01.2, M01.3, M01.4, M01.5, M01.8, M01.9, M02.6, M02.7, M08.4, M17.2, M17.4, M17.8, M17.9, X40.1, X40.2, X40.3, X40.5, X40.6, X41.1, X41.2, X42.1
**COPD**	68, 148, 152, 312, 794, 998, 1001, 1382, 1446, 1934, 2581, 3163, 3243, 3480, 4519, 4899, 5710, 5798, 5909, 5978, 7092, 7884, 9043, 9389, 9520, 9876, 10403, 10802, 10863, 10980, 11072, 11101, 11150, 11287, 12166, 14798, 15157, 15626, 16287, 16410, 17359, 18621, 18792, 19400, 19434, 19721, 20198, 21145, 21492, 22795, 22905, 23492, 24248, 24316, 24800, 25603, 26018, 26306, 27819, 28743, 28755, 29166, 29273, 29457, 29669, 30653, 31886, 33450, 34202, 34215, 37247, 37371, 37959, 38074, 40159, 40788, 41137, 42258, 42313, 43362, 44525, 45089, 45770, 45777, 45998, 46578, 48593, 49794, 50396, 54893, 55391, 56860, 59263, 60188, 61118, 61513, 63479, 64890, 65733, 65916, 66043, 66058, 67040, 68066, 68662, 70787, 71370, 73100, 92955, 93153, 93568	J40, J41, J42, J43, J440, J441, J448, J449, J20
**Diabetes**	758, 1407, 1549, 10418, 10692, 12455, 12640, 12736, 17545, 17858, 17859, 18143, 18209, 18219, 18230, 18264, 18278, 18387, 18390, 18425, 18496, 18642, 18683, 18777, 21983, 22871, 22884, 24423, 24458, 24836, 25591, 25627, 26054, 30294, 30323, 32627, 34268, 34450, 35288, 35385, 36633, 37806, 38161, 39070, 40682, 40837, 41049, 42729, 42762, 42831, 43227, 43921, 44779, 44982, 45913, 45914, 45919, 46150, 46301, 46850, 46917, 47315, 47321, 47409, 47582, 47649, 47650, 47816, 47954, 48192, 49074, 49146, 49554, 49655, 49869, 49949, 50225, 50527, 50813, 51756, 51957, 53392, 54008, 54899, 55075, 55239, 56268, 57278, 58604, 59253, 59725, 60107, 60208, 60699, 60796, 61071, 61344, 61829, 62107, 62209, 62352, 62613, 62674, 63017, 63690, 64571, 64668, 65267, 65704, 66145, 66872, 66965, 67905, 68105, 68390,	E10, E11, E12, E13, E14, G590, G632, H280, H360, M142, N083, O240, O241, O242, O243

	69676, 69993, 70316, 70766, 85991, 91646, 91942, 91943, 93468, 93727, 93878, 95343, 95351, 95992, 96235, 97446, 97474, 97894, 98616, 98723	
**Dyslipidemia**	339, 340, 637, 2091, 3386, 3484, 5791, 6243, 7447, 8762, 9936, 10783, 10899, 12439, 12569, 13228, 16085, 16290, 16306, 16534, 18708, 26019, 30335, 32244, 33694, 34146, 34224, 34825, 36806, 37273, 39147, 39783, 42765, 50923, 51023, 52992, 53091, 54499, 55855, 59095, 59564, 66240, 67948, 68741, 70793, 71747, 93761, 95952, 97166, 97890, 97989	E78
**Fatty liver**	10572, 10691, 98148, 103466, 103706	K70.0, K75.8, K76.0
**Heart failure**	398, 884, 1223, 2062, 2906, 4024, 5141, 5255, 5695, 5942, 8464, 8966, 9524, 9913, 10079, 10154, 11424, 12550, 13189, 17278, 18853, 19066, 21837, 22262, 23481, 23707, 24503, 26242, 27884, 27964, 32671, 46672, 51214, 52127, 57987, 62718, 68766, 72668, 94870, 104275	I110, I132, I130, I50
**Hypertension**	204, 799, 1894, 3425, 3712, 3979, 4372, 4668, 6702, 7057, 7329, 8296, 8732, 8857, 10818, 11056, 13188, 15106, 15377, 16059, 16173, 16292, 16565, 18057, 18482, 18590, 18765, 19070, 20497, 21660, 21826, 21837, 22333, 25371, 27511, 28684, 29310, 30770, 31341, 31387, 31464, 31755, 31816, 32423, 32976, 34744, 37086, 39649, 42229, 43664, 43935, 44350, 44549, 50157, 51635, 52127, 52427, 52621, 57288, 57987, 59383, 60655, 61166, 61660, 62432, 62718, 63000, 63164, 63466, 66567, 67232, 68659, 69753, 72030, 72668, 73293, 73586, 83473, 85944, 93055, 95334, 95359, 96743, 97533	I10, I11, I12, I13, I15
**Ischemic stroke**	569, 3149, 5185, 5363, 5602, 6155, 8837, 9985, 10504, 15019, 15252, 16517, 23671, 24446, 25615, 26424, 27975, 33543, 34758, 36717, 40758, 47642, 53745, 57495, 91627, 94482	I63.4, I69.3<br>I63.0, I63.8<br>I63.1, I63.5<br>I63.2, I63.9<br>I63.3
**Myocardial Infarction**	241, 1677, 1678, 2491, 3704, 5387, 7783, 8935, 9507, 10562, 12139, 12229, 13571, 14658, 14897, 14898, 15661, 17872, 18842, 23708, 23892, 24126, 26972, 26975, 28736, 29553, 29643, 29758, 30330, 30421, 32272, 32854, 34803, 35119, 36423, 37657, 38609, 40429, 41221, 41835, 45809, 46017, 46112, 46166, 46276, 52705, 55401, 59032, 59189, 59940, 61670, 62626, 63467, 68357, 68748, 69474, 72562, 96838	I21, I22, I23, I241, I252
**Peripheral arterial disease**	1517, 1826, 2065, 2066, 2760, 2761, 3530, 3715, 3778, 4317, 4325, 4539, 5414, 5650, 5702, 5943, 6256, 6308, 6356, 6617, 6827, 6853, 7111, 8801, 9099, 9119, 9204, 9561, 10500, 10827, 11624, 11766, 12331, 12735, 14796, 14797, 14895, 15272, 15302, 15532, 16260, 16363, 17336, 18030, 18038, 18060, 18816, 19155, 20657, 20892, 21927, 22016, 22834, 23352, 23497, 23871, 24097, 24229, 24327, 24677, 24692, 25954, 27494, 27580, 28030, 28119, 28125, 28166, 28616, 28777, 28894, 29112, 30484,	I731, I738, I739, I743, I744, I745, L50, L50.1, L50.2, L50.3, L50.4, L50.5, L50.6, L50.8, L50.9, L51, L51.1, L51.2, L51.3, L51.4, L51.5, L51.6, L51.8, L51.9, L52, L52.1, L52.2, L52.8, L52.9, L53, L53.1, L53.2, L54.1, L54.2,

	30989, 31053, 31338, 32492, 32634, 33555, 34037, 34152, 34153, 34638, 35399, 36065, 36136, 36443, 36952, 37787, 37806, 38907, 38921, 39039, 39437, 39776, 39877, 40302, 40397, 40619, 40732, 41768, 41823, 42115, 42465, 42640, 42645, 43648, 43651, 44250, 44430, 44835, 45428, 47562, 47835, 48700, 48755, 48939, 49273, 49319, 50894, 51211, 51331, 52289, 52342, 52357, 52695, 52869, 53580, 53634, 53675, 54071, 54212, 54865, 54899, 55402, 55554, 55825, 55877, 56803, 56919, 57793, 59602, 60212, 60465, 60693, 60699, 61255, 61256, 61974, 62775, 62818, 62866, 63238, 63280, 63357, 63368, 63396, 63605, 64446, 64555, 64798, 65025, 65286, 65669, 65692, 66437, 66804, 66820, 66869, 66879, 66917, 66930, 67083, 67401, 67818, 67982, 68141, 68320, 68412, 68698, 69124, 69232, 69519, 70448, 70922, 71041, 71860, 72448, 72491, 72632, 73822, 73961, 81445, 93468, 95573, 96255, 96809, 97606, 98174	L54.4, L54.8, L54.9, L58, L58.1, L58.2, L58.3, L58.4, L58.5, L58.6, L58.7, L58.8, L58.9, L59, L59.1, L59.2, L59.3, L59.4, L59.5, L59.6, L59.7, L59.8, L59.9, L60, L60.1, L60.2, L60.3, L60.4, L60.8, L60.9, L62, L62.1, L62.2, L62.8, L62.9, L63.1, L63.2, L63.3, L63.5, L65, L65.1, L65.2, L65.3
**Obstructive Sleep apnea**	2506, 7603, 8148, 20438, 20748, 23779, 24755, 36301, 38059, 38294, 48539	E662, G473
**Venous Thromboembolism**	824, 1224, 3392, 3576, 3585, 5503, 8769, 15382, 20161, 20676, 22006, 22038, 25478, 27284, 28309, 31390, 32002, 36568, 37947, 38099, 39344, 41180, 42506, 43555, 48920, 49715, 54205, 55661, 55883, 55885, 57100, 58023, 58166, 61366, 61760, 64467, 65855, 70455, 70467, 84404, 91282, 94496, 95077, 97808, 97853, 98526, 100103, 101733, 104342, 105403, 106482, 106850, 107851	I26, I63.6, I67.6, I80.1, I80.2, I81, I82.0, I82.2, I82.3
**Medication**	**Production code**	
**Aspirin**	3, 16, 34, 254, 306, 377, 381, 393, 395, 430, 434, 484, 645, 657, 685, 1049, 1137, 1902, 2047, 2105, 2607, 2628, 2986, 3155, 3309, 3386, 3726, 4679, 5288, 6006, 6007, 6226, 6666, 6696, 7516, 7518, 7520, 7539, 8185, 8186, 8645, 9044, 9129, 9144, 9301, 9432, 9939, 10031, 10305, 10310, 11326, 11951, 11961, 11977, 12047, 12964, 12976, 12992, 13882, 14517, 15364, 15367, 15779, 16184, 16611, 17180, 17456, 17704, 17920, 17926, 18030, 18217, 18261, 18329, 19189, 19255, 20127, 20650, 20840, 21067, 21380, 21382, 21770, 21921, 22138, 22232, 22305, 22618, 22776, 23488, 23593, 23841, 23878, 23932, 24025, 24309, 24622, 24828, 24960, 25211, 25335, 25433, 25718, 26967, 27435, 28784, 28810, 29054, 29759, 29848, 30022, 30920, 31001, 31210, 31211, 31499, 31858, 31870, 31894, 31938, 31953, 31954, 31956, 32036, 32154, 32178, 32210, 32314, 32728, 32992, 33139, 33293, 33317, 33320, 33656, 33662, 33668, 33676, 34233, 34309, 34385, 34386, 34434, 34485, 34611, 34666, 34762, 34796, 34797, 34942, 35967, 36521, 36543, 37541, 39738, 40144, 40381, 41512, 41569, 41594, 41766, 42218, 43060, 43434, 43679, 43709, 43806, 44639, 45643, 45840, 45851, 46925, 47937, 47992, 48000, 48021, 48165, 48974, 49060, 49220, 49685, 49799, 50166, 50555, 50926, 50949, 51474, 51561, 52044, 52280, 52618, 52856, 52905, 53178, 53325, 53622, 53711, 53791, 53804, 53816, 54284, 54353, 54430, 54526, 54565, 54734, 54997, 55230, 55579, 56007, 56503, 56736, 56883, 56995, 56996, 57057, 57585, 58112, 58331, 59021, 59244, 59253, 59728, 59791, 60127, 60278, 60693, 60694, 60777, 62334, 62430, 63603, 63658, 63658, 63683, 64071, 65027, 66171, 66345, 66546, 66563, 66861, 67124, 67160, 67362, 67521, 67754, 67858, 68051, 68051, 68752, 68764, 69216, 70549, 70841, 71078, 71192, 71663, 71663, 71676, 71821	

**Clopidogrel**	489, 836, 38349, 38998, 40913, 42750, 45905, 46891, 52761, 53751, 54700, 55161, 56807, 57036, 58347, 58448, 59904, 62855, 62978, 63450, 65909, 67037
**Digoxin**	36, 94, 792, 2302, 2511, 3181, 3286, 3705, 9522, 16366, 20844, 20944, 25238, 27523, 29282, 33080, 33274, 33612, 33675, 34017, 34023, 34024, 34327, 34328, 34519, 34948, 40245, 42989, 42990, 43577, 47813, 48587, 54117, 61258, 62678, 62679, 62680, 63286, 64045, 67227, 68261, 69470, 70125, 71439
**DOAC**	39444, 39503, 39755, 42474, 46632, 46678, 47566, 53740, 54066, 58594, 39119, 39639, 47207, 47353, 47925, 48134, 48966, 54451, 56289, 56640, 62150, 64500
**Warfarin**	45, 61, 833, 1781, 6262, 8466, 8467, 13348, 17965, 23078, 30202, 30203, 31511, 31937, 33711, 34019, 34086, 34087, 34088, 34095, 34299, 34416, 34417, 34418, 34517, 34526, 34576, 34691, 34758, 34864, 34918, 36099, 38041, 38044, 39866, 40143, 43407, 43408, 43409, 43655, 44866, 47944, 48070, 48869, 50000, 51484, 51496, 51509, 53745, 53752, 54892, 54946, 55316, 56314, 57032, 58519, 58787, 58962, 59400, 59578, 60589, 60949, 62309, 62310, 63071, 65285, 65496, 65746, 66286, 66570, 68591, 68667, 68795, 69128, 71196, 71386, 72040

Table S.2. Multivariate predictors of all-cause mortality, heart failure, myocardial infarction and ischemic stroke hospitalization in the complete-case sensitivity analysis

	All-cause mortality		Heart failure hospitalization		Myocardial infarction hospitalization		Ischemic stroke hospitalization		Bleeding hospitalization	
	**HR**	**95%CI**	**HR**	**95%CI**	**HR**	**95%CI**	**HR**	**95%CI**	**HR**	**95%CI**
**Obese**	0.69	0.67-0.71	1.2	1.12-1.28	0.91	0.83-1.00	0.96	0.88-1.06	0.87	0.81-0.92
**Overweight**	0.72	0.70-0.74	0.95	0.89-1.01	0.94	0.86-1.02	1.05	0.97-1.14	0.92	0.87-0.98
**Underweight**	1.91	1.81-2.01	0.91	0.75-1.09	1.28	1.01-1.61	1.07	0.86-1.34	0.89	0.75-1.06
ref Normal weight										
**Age** (per year)	1.06	1.06-1.06	1.04	1.03-1.04	1.04	1.03-1.04	1.05	1.05-1.05	1.02	1.02-1.02
**Female Sex**	0.87	0.85-0.89	0.89	0.85-0.94	0.95	0.88-1.02	1.34	1.25-1.44	0.81	0.77-0.85
**White ethnicity**	1.00	0.93-1.08	0.67	0.59-0.76	0.84	0.69-1.03	0.79	0.65-0.96	1.01	0.87-1.16
**Most Deprived quintile**	1.11	1.08-1.13	1.09	1.04-1.16	1.06	0.98-1.15	1.04	0.96-1.12	0.95	0.90-1.00
**Current or Ex-Smokers**	1.19	1.16-1.22	1.10	1.04-1.16	1.15	1.06-1.24	1.03	0.95-1.10	1.00	0.95-1.05
**Alcohol related disorder**	1.50	1.41-1.59	1.06	0.93-1.22	1.06	0.86-1.30	1.24	1.00-1.53	1.41	1.25-1.58
**Heart failure**	1.70	1.65-1.74	3.48	3.31-3.66	1.32	1.22-1.43	0.95	0.87-1.04	1.13	1.07-1.2
**Hypertension**	1.02	0.99-1.05	1.13	1.06-1.2	1.25	1.15-1.37	1.26	1.16-1.37	1.22	1.15-1.29
**Ischemic stroke**	1.34	1.28-1.40	0.96	0.86-1.07	1.02	0.88-1.18	3.22	2.93-3.54	1.13	1.02-1.24
**Myocardial infarction**	1.17	1.14-1.21	1.46	1.38-1.54	2.84	2.63-3.07	1.04	0.94-1.14	0.99	0.93-1.06
**VTE**	1.25	1.20-1.30	1.07	0.99-1.17	1.04	0.91-1.19	0.90	0.79-1.03	1.12	1.03-1.22
**PAD**	1.36	1.32-1.41	1.24	1.15-1.33	1.42	1.29-1.58	1.14	1.01-1.28	1.10	1.01-1.19
**Diabetes Mellitus**	1.34	1.3-1.37	1.37	1.3-1.45	1.46	1.35-1.57	1.24	1.15-1.34	1.12	1.06-1.18
**CKD**	2.29	2.09-2.51	0.98	0.76-1.25	2.04	1.56-2.66	1.02	0.67-1.55	1.87	1.53-2.3
**Dyslipidemia**	0.88	0.86-0.91	0.95	0.90-1.00	1.08	1.00-1.17	1.01	0.94-1.09	1.02	0.97-1.07
**Obstructive sleep apnea**	1.02	0.91-1.14	1.25	1.05-1.48	0.82	0.59-1.14	0.79	0.53-1.16	1.28	1.08-1.52
**COPD**	1.20	1.17-1.23	1.17	1.11-1.23	1.09	1.01-1.17	0.96	0.89-1.03	1.07	1.02-1.12
**Fatty liver**	1.35	1.14-1.59	1.11	0.79-1.55	1.64	1.07-2.51	1.14	0.64-2.03	1.57	1.18-2.09
**Asthma**	1.12	1.09-1.15	1.09	1.02-1.16	1.14	1.03-1.25	0.85	0.77-0.94	1.09	1.03-1.16

Legend: HR – Hazard ratio; CI – confidence interval; VTE – venous thromboembolism; PAD – peripheral artery disease; CKD – chronic kidney disease; COPD – chronic obstructive pulmonary disease.

Figure S.1. Kaplan-Meier survival curves for heart failure hospitalization (long follow-up)

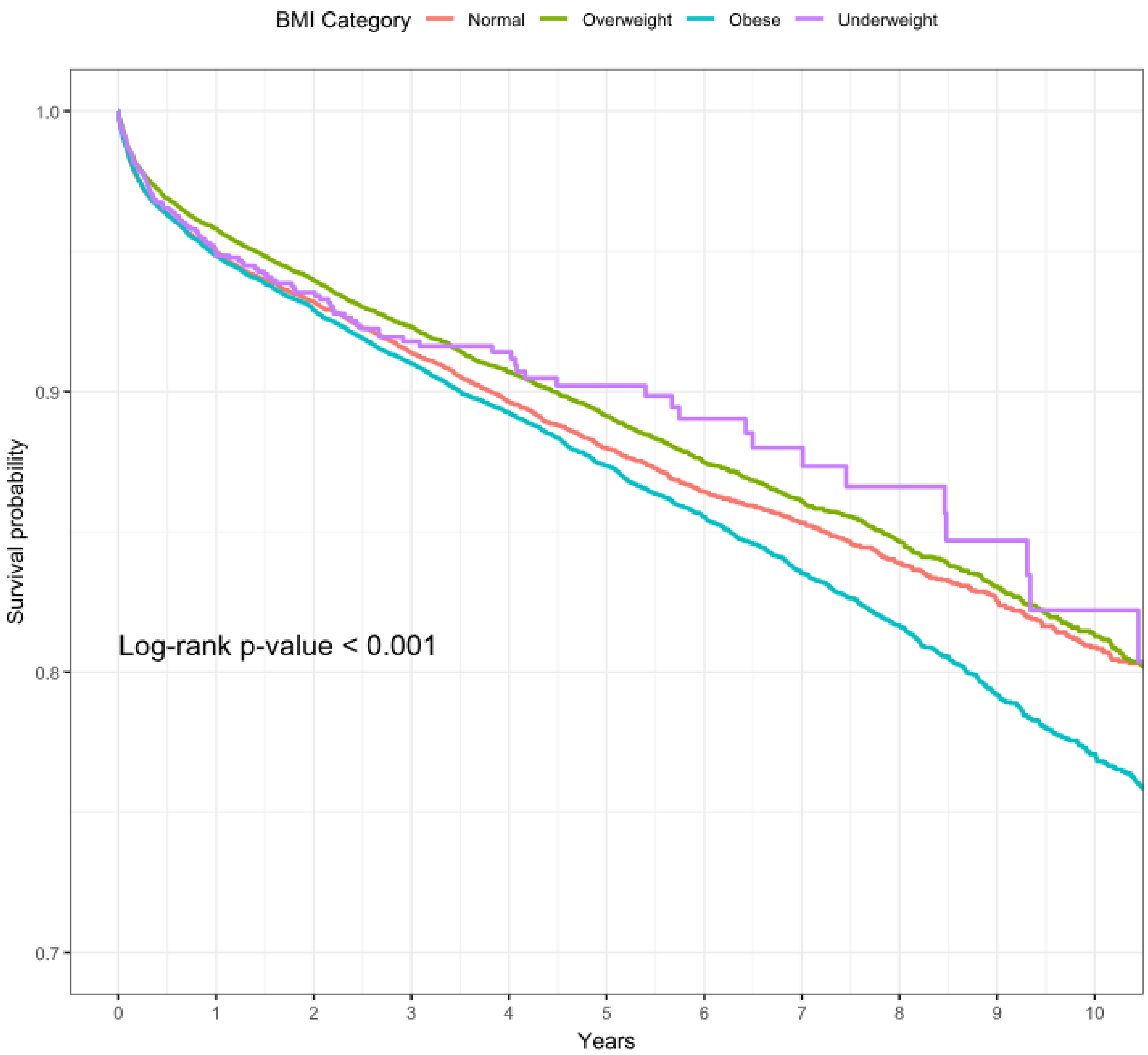

Number at risk

Normal	21731	13958	11169	8872	6986	5420	4214	3235	2480	1821	1311
Overweight	25048	18478	15488	12743	10366	8333	6679	5238	4090	3052	2236
Obese	22268	16931	14275	11821	9681	7676	6138	4751	3544	2590	1859
Underweight	2472	1087	787	573	401	282	200	133	101	77	50

Figure S.2. Kaplan-Meier survival curves for myocardial infarction hospitalization (5-year follow-up)

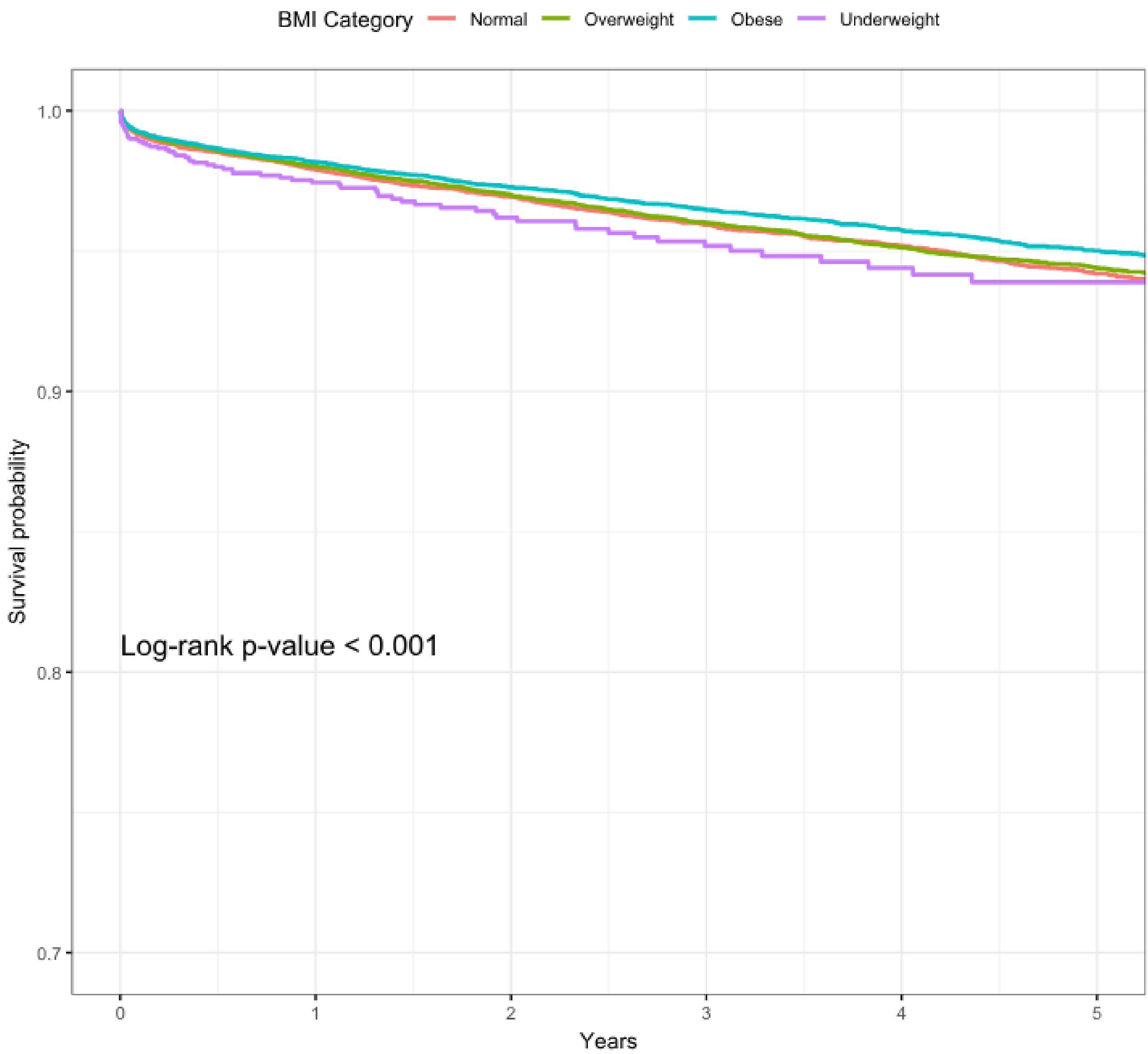

Number at risk

Normal	21731	14188	11355	9031	7139	5532
Overweight	25048	18758	15747	12974	10570	8512
Obese	22268	17406	14749	12280	10106	8054
Underweight	2472	1093	787	578	400	282

Figure S.3. Kaplan-Meier survival curves for myocardial infarction hospitalization (long follow-up)

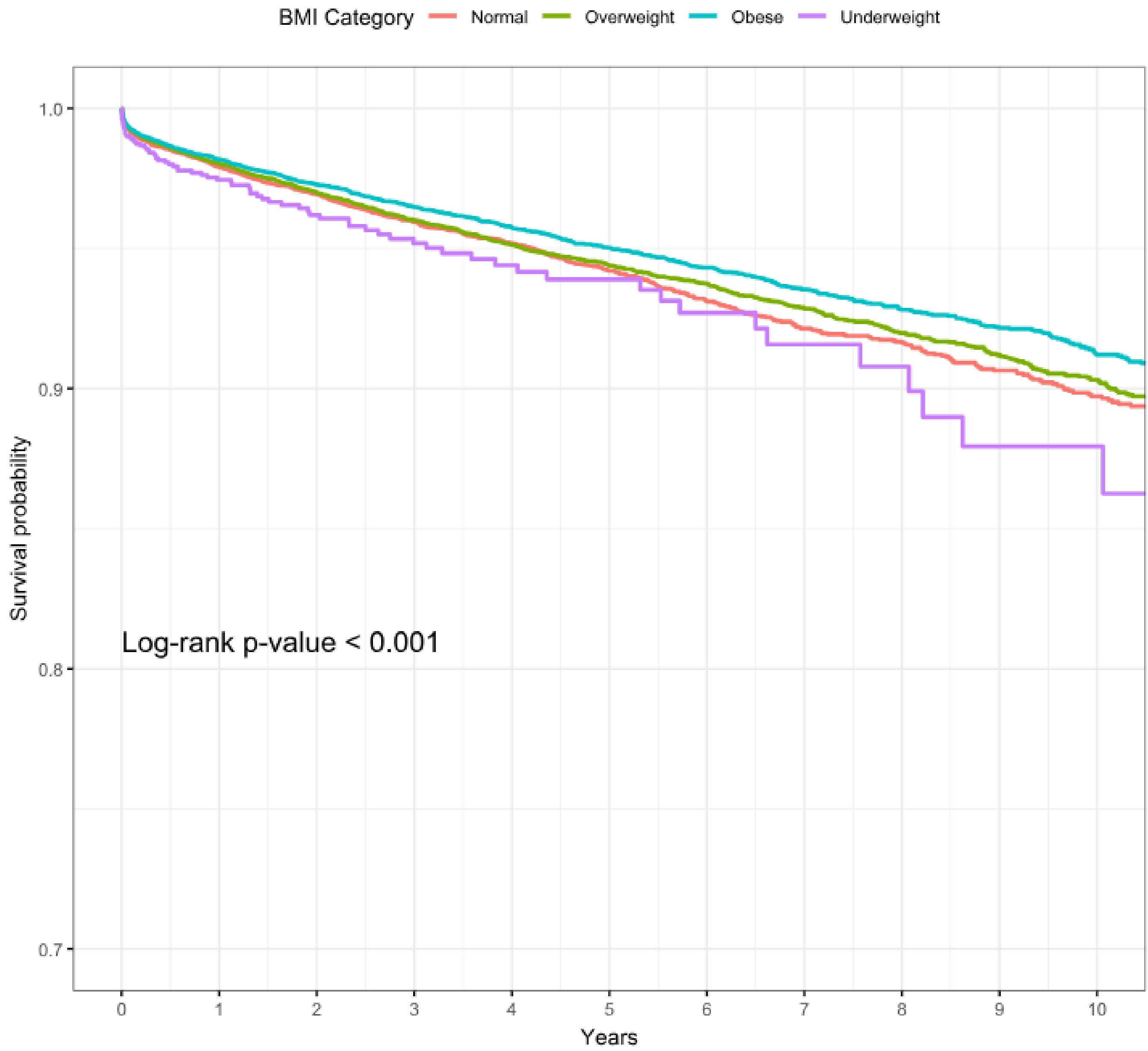

Number at risk

Normal	21731	14188	11355	9031	7139	5532	4289	3279	2518	1845	1338
Overweight	25048	18758	15747	12974	10570	8512	6824	5344	4149	3088	2276
Obese	22268	17406	14749	12280	10106	8054	6471	5006	3729	2771	1976
Underweight	2472	1093	787	578	400	282	198	133	103	79	53

Figure S.4. Kaplan-Meier survival curves for Ischemic stroke hospitalization (5-year follow-up)

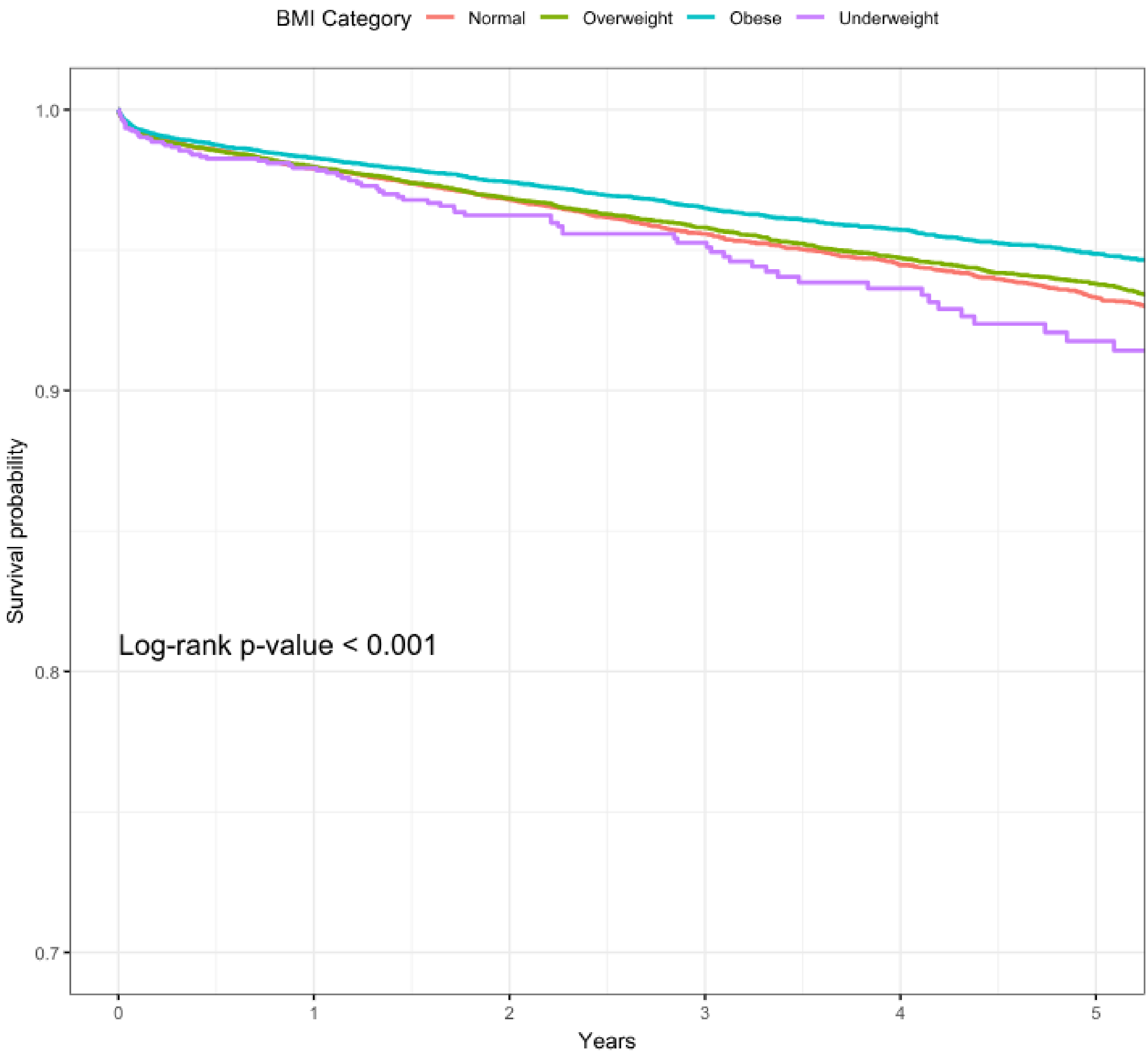

Number at risk

	0	1	2	3	4	5
Normal	21731	14205	11368	9033	7125	5510
Overweight	25048	18761	15744	12973	10553	8499
Obese	22268	17443	14776	12280	10106	8044
Underweight	2472	1107	798	583	402	281

Figure S.5. Kaplan-Meier survival curves for Ischemic stroke hospitalization (long follow-up)

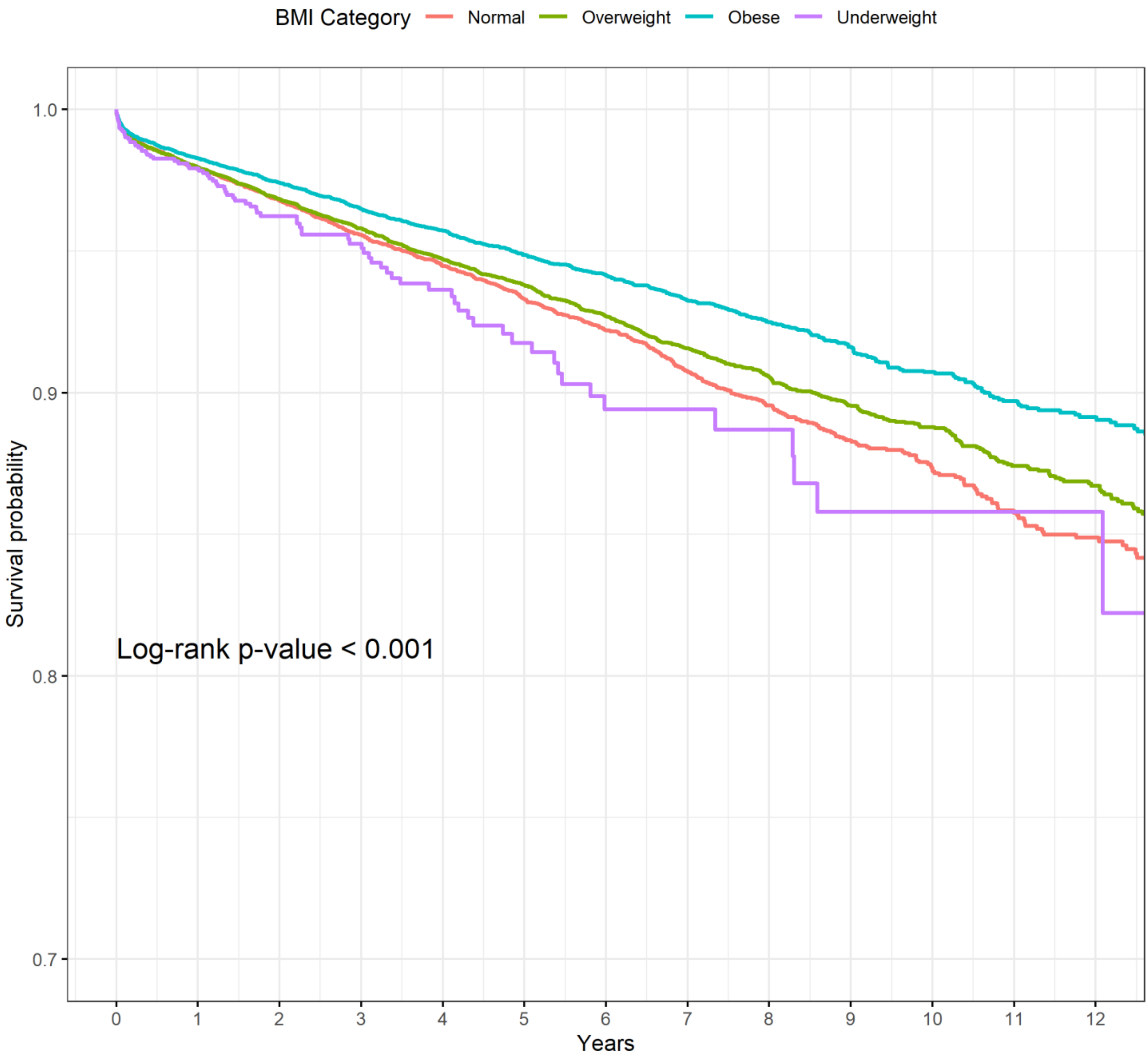

Number at risk

BMI Category	0	1	2	3	4	5	6	7	8	9	10	11	12
Normal	21731	14205	11368	9033	7125	5510	4277	3268	2516	1853	1336	964	672
Overweight	25048	18761	15744	12973	10553	8499	6799	5343	4169	3109	2284	1618	1160
Obese	22268	17443	14776	12280	10106	8044	6475	5025	3753	2797	2006	1436	991
Underweight	2472	1107	798	583	402	281	197	133	100	79	53	39	24

Years

Figure S.6. Kaplan-Meier survival curves for bleeding hospitalization (5-year follow-up)

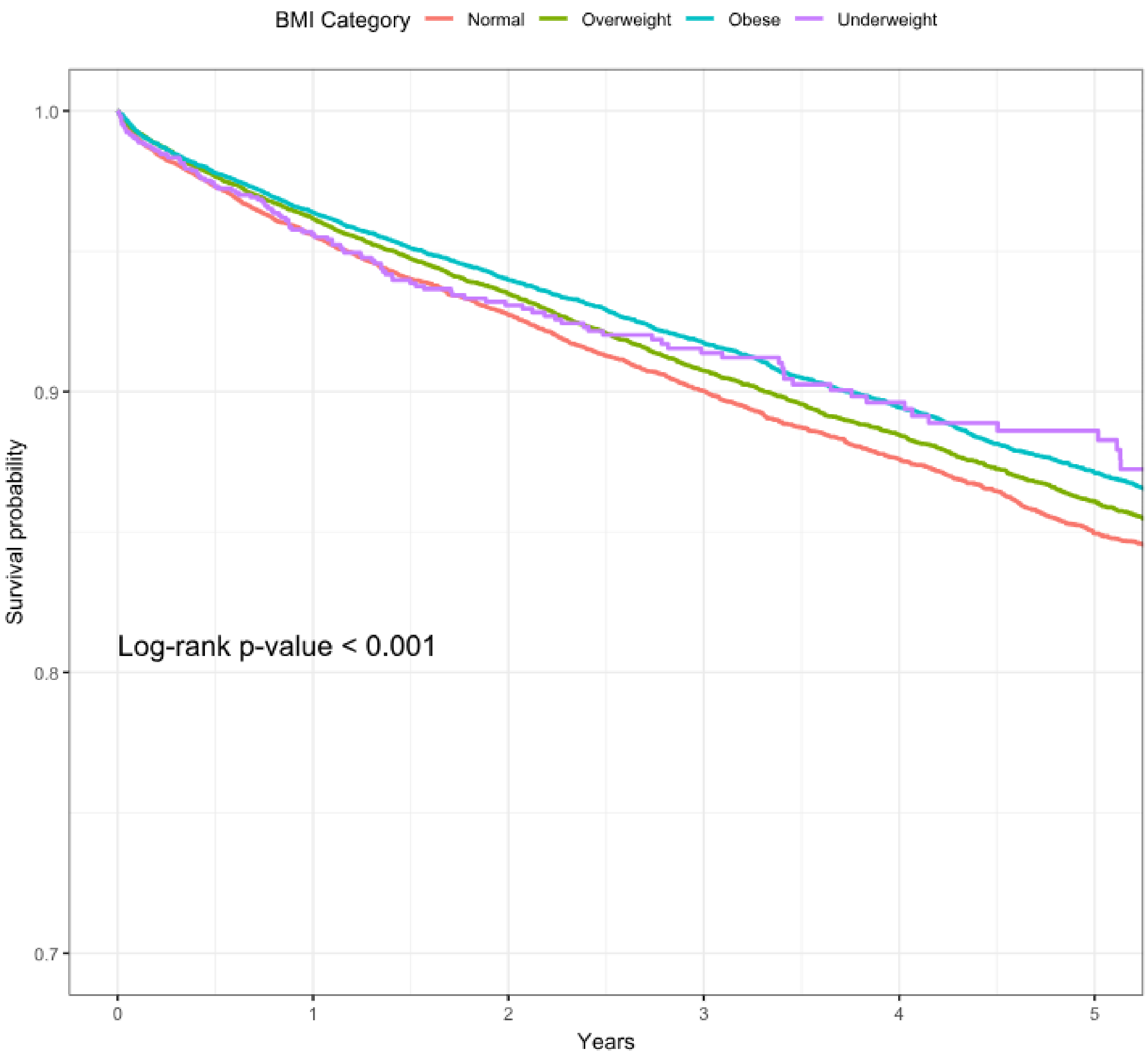

Number at risk

Normal	21731	13884	10942	8581	6690	5092
Overweight	25048	18409	15219	12369	9933	7873
Obese	22268	17079	14271	11707	9481	7435
Underweight	2472	1078	762	554	381	268

Figure S.7. Kaplan-Meier survival curves for bleeding hospitalization (long follow-up)

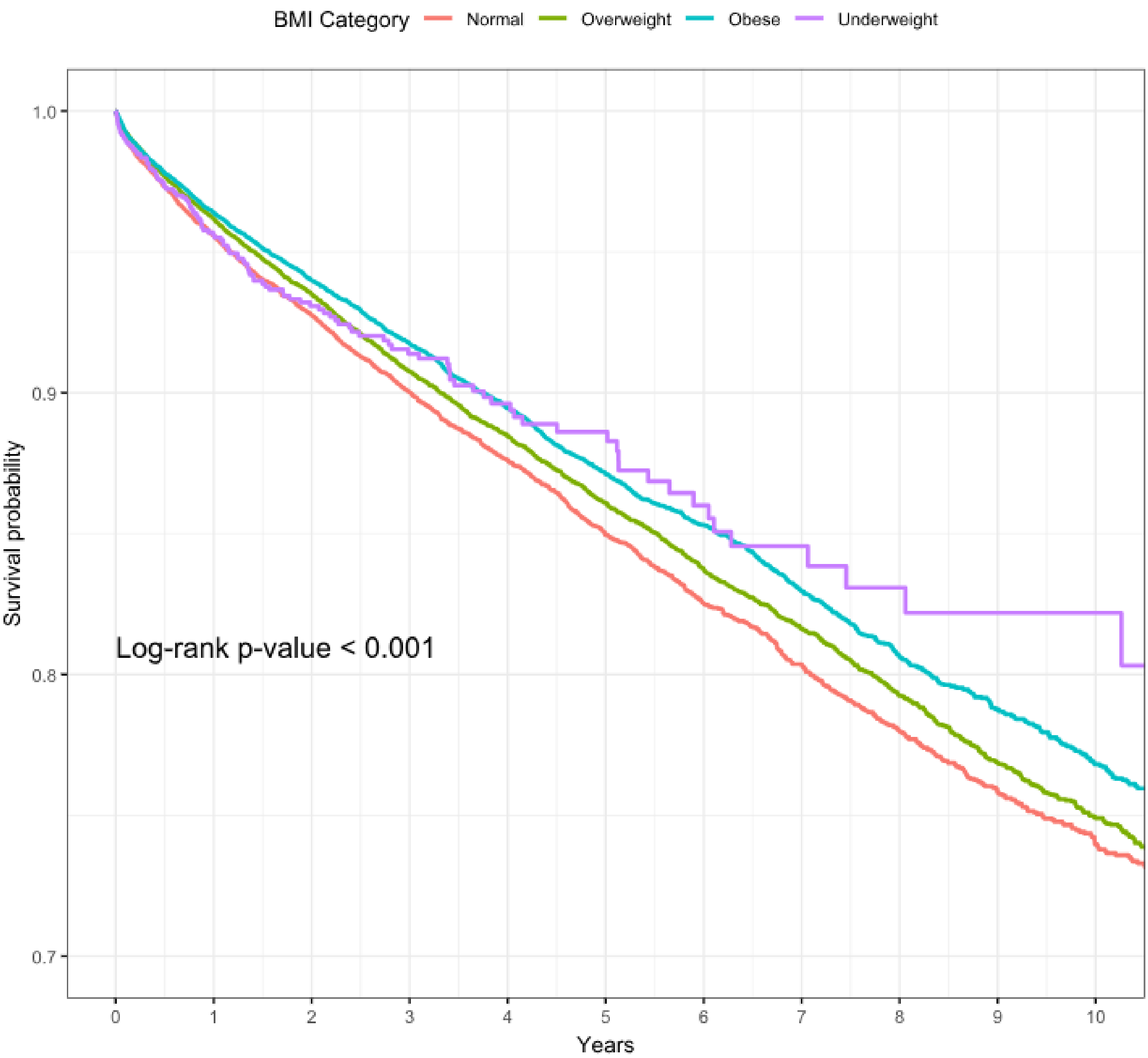

Number at risk

Normal	21731	13884	10942	8581	6690	5092	3905	2950	2219	1609	1161
Overweight	25048	18409	15219	12369	9933	7873	6208	4825	3701	2701	1950
Obese	22268	17079	14271	11707	9481	7435	5945	4547	3336	2440	1745
Underweight	2472	1078	762	554	381	268	186	120	93	71	47

Figure S.8. Spline Curves for myocardial infarction hospitalization

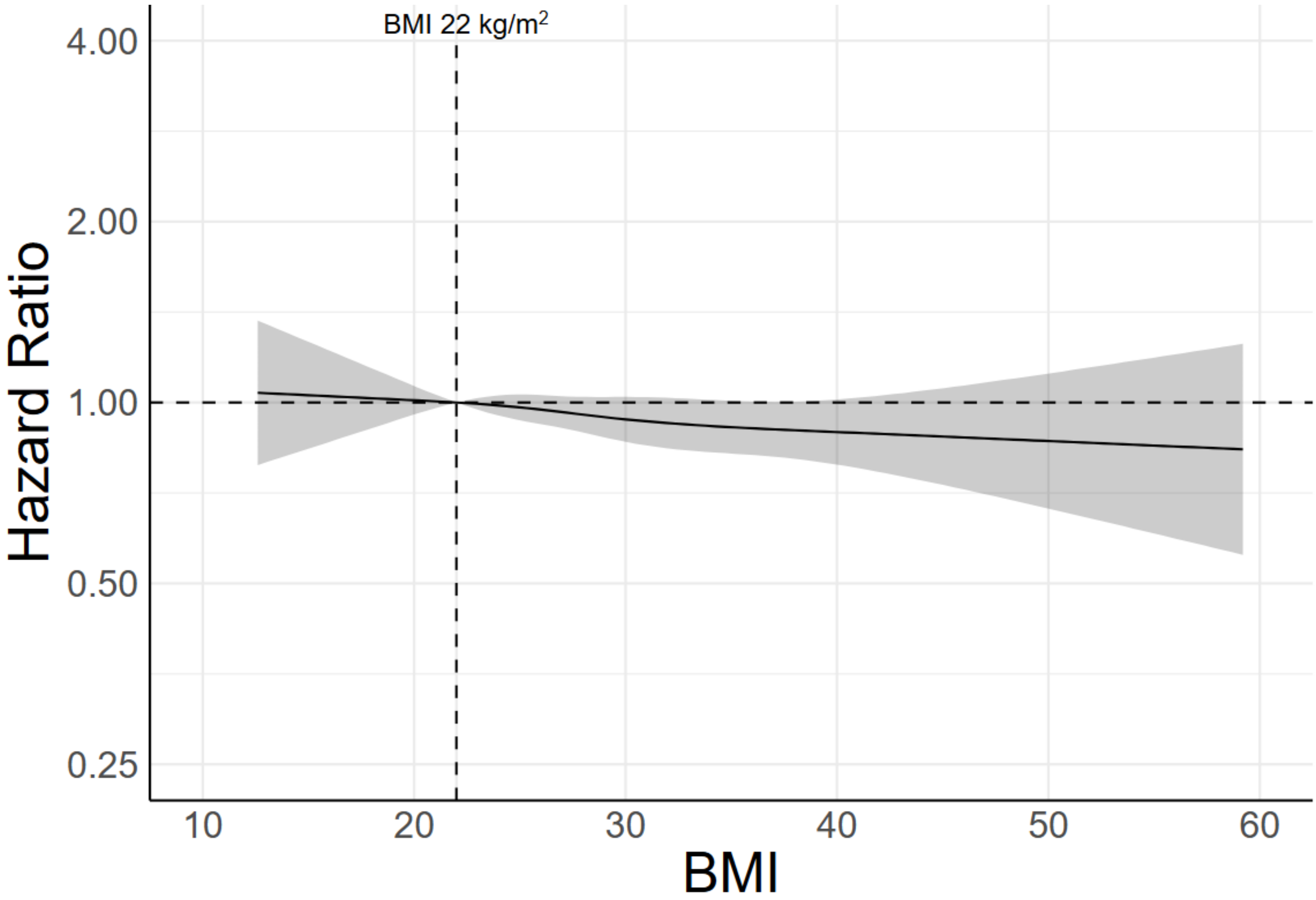

Figure S.9. Spline Curves for ischemic stroke hospitalization

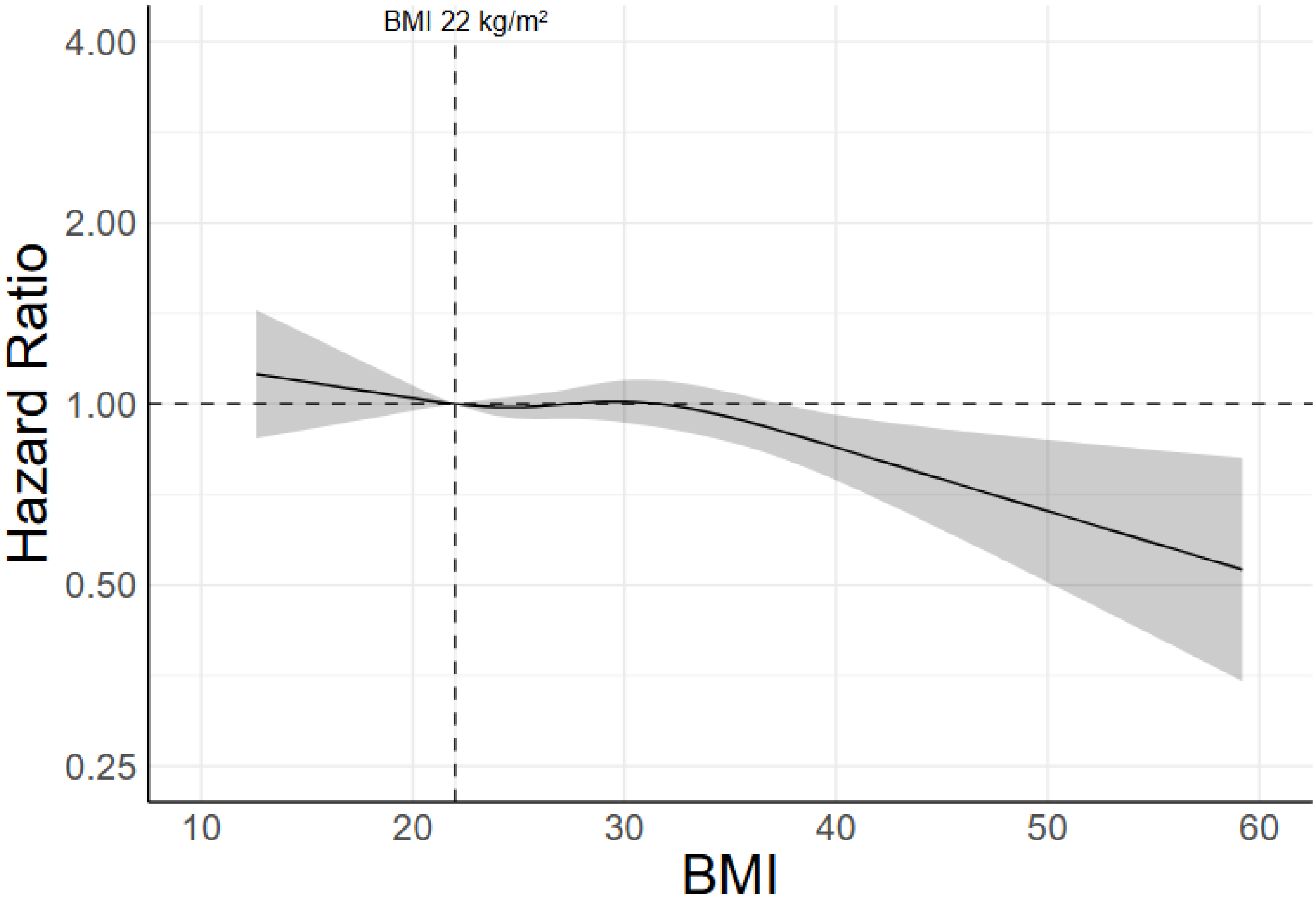

Figure S.10. Spline Curves for bleeding hospitalization

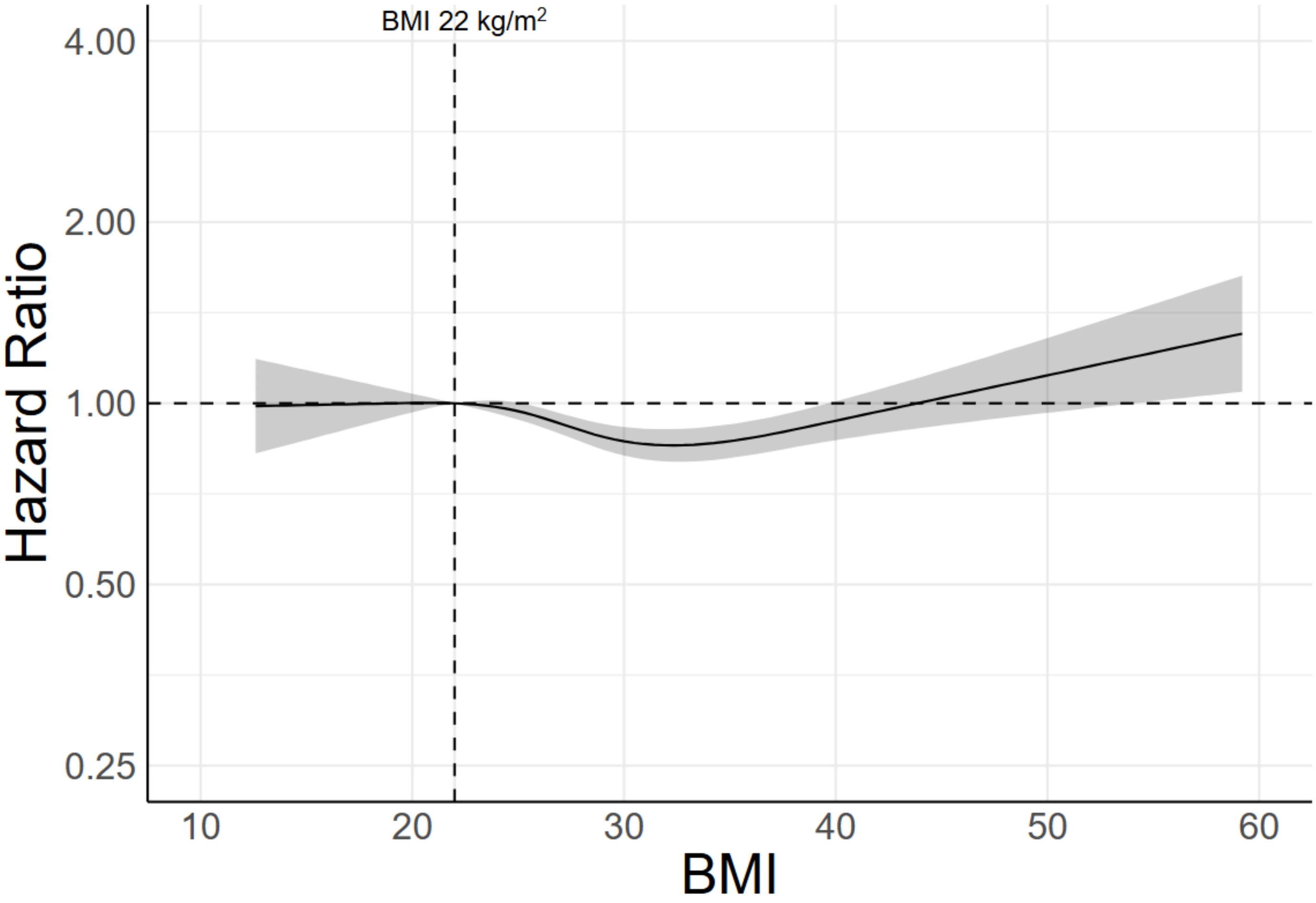

Supplement: Supplementary file 1 [file mmc1.pdf]
